# Simple Method for Controlling Gold Nanocluster Size in Mesoporous Silica: SBA-11

**DOI:** 10.3390/molecules30092035

**Published:** 2025-05-02

**Authors:** Tariq Aqeel, Ali Bumajdad

**Affiliations:** 1 Department of Science, College of Basic Education, The Public Authority for Applied Education and Training (PAAET), P.O. Box 23167, Safat 13092, Kuwait; 2 Chemistry Department, Faculty of Science, Kuwait University, P.O. Box 5969, Safat 13060, Kuwait; a.bumajdad@ku.edu.kw

**Keywords:** controlled, nanoclusters, gold, mesoporous, methylene blue, photocatalysis, sunlight, XPS, UV-vis

## Abstract

Mesoporous silica containing Au nanoclusters has been widely used in various fields owing to its desirable properties and functionality. This work introduces a facile method to control the size of Au nanoclusters within silica hosts. This was achieved by applying a reducing gas at a controlled temperature to obtain a narrow or broad Au cluster size distribution inside the silica host. The Au nanoclusters and their silica hosts were characterized through X-ray diffraction, high-resolution transmission electron microscopy, energy-dispersive X-ray spectroscopy, ultraviolet-visible spectroscopy, N_2_ sorption analysis, and X-ray photoelectron spectroscopy. The average size of the Au nanoclusters was 1.3 nm in the narrow-distribution sample. In comparison, the wide-distribution sample exhibited two cluster populations: 1–2 nm inside the pores and 5–30 nm outside the pores. Methylene blue (M.B.) photocatalysis was conducted under direct sunlight to evaluate the catalytic activity of these materials. Sample 1–Au–SBA-11, containing Au clusters averaging 1.3 nm, achieved substantial M.B. degradation within 90 min—half the time required by the 2–Au–SBA-11 sample. Such Au clusters of different size ranges have potential applications across diverse fields.

## 1. Introduction

Mesoporous silica and Au nanoclusters have been widely utilized in recent decades. Their applications include catalysis [1], detection and selective removal of heavy metals from water [2], detection of bisphenol [3], and development of photonic devices [4] and flexible sensors [5]. Furthermore, mesoporous silica is considered a biocompatible substance [6]; therefore, it is used extensively in biological research for the early detection of viruses [7] and cancer [8], noninvasive theranostic approaches [9], neuromorphic switching [10], tumor cell targeting [11,12,13,14], photothermal treatments [15], cardiac tissue engineering [16], development of theranostic devices [17], detection of pathogens, compounds, and toxins such as lectin [18], magnetic resonance imaging [6], and drug delivery [19]. More recently, it has been utilized in drug delivery systems to target specific organs and overcome biological barriers [19,20,21]. The shape and size of both mesoporous silica and nanoclusters are critical in delivery systems [19]. For instance, small nanoclusters (<200 nm) more readily penetrate the gastrointestinal (GI) mucus barrier than larger ones. Additionally, in brain-targeted drug delivery, smaller Au clusters (3–40 nm) exhibit faster in vivo permeation than larger clusters [20]. Furthermore, long nanorods are absorbed more efficiently than spherical clusters, but remain in the GI tract longer and are harder to eliminate. Additionally, the porosity of mesoporous silica facilitates delayed drug release following enzymatic degradation [20]. Altering the shape and aspect ratio of Au nanoclusters influences their surface plasmon resonance range [11,22].

Nanoclusters can be introduced into mesoporous silica in various ways, such as anchoring a functional group onto the surface of mesoporous silica [23,24] or first incorporating the metal of interest into a surfactant or in micelles [25,26] and then synthesizing the silica source around them. However, both of these general strategies suffer from limitations. First, the functional groups must be kept in the final product. Otherwise, the metallic nanoclusters may leach out. This also limits the accessibility to the metal of interest [27] and dramatically decreases the surface area of the host. The functional groups also limit the use of the final product at elevated temperatures of 500 °C or higher, because they would burn off at such temperatures, causing the metallic nanoclusters to leach out during catalysis. In addition, these methods add another step to the synthesis procedure. The second route using surfactant or micelles also faces limitations. First, some metals cannot be incorporated into the surfactants; thus, the selection of metals is limited. Second, this also adds a lengthy step to the synthesis method. This study avoids these limitations by developing a simple strategy for producing mesoporous silica containing Au clusters that are uniform in size and shape or that have different sizes and shapes. Researchers can select either pathway depending on the intended application. In addition, a photocatalytic experiment was conducted to compare the activities of these materials on the basis of their synthetic pathways.

## 2. Results and Discussion

### 2.1. Narrow-Angle XRD

**Figure 1:**
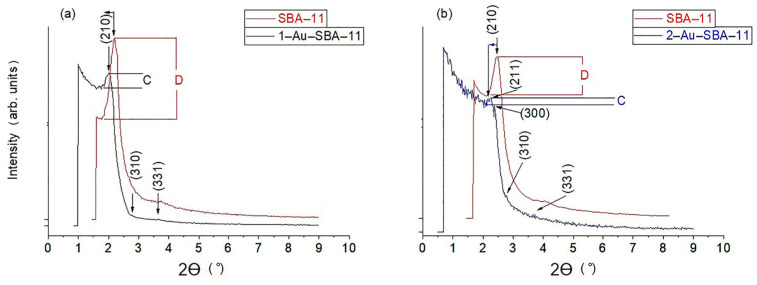
Narrow-angle XRD patterns of (**a**) 1–Au–SBA-11 and (**b**) 2–Au–SBA-11 in comparison with those of pristine SBA-11.

Figure 1 compares the narrow-angle XRD patterns of 1–Au–SBA-11 (a) and 2–Au–SBA-11 (b) with that of pristine SBA-11 (shown in red). Both samples show a shift of the main Bragg peak (210) to a lower angle. This shift resulted from the second heat treatment associated with the conversion of the Au precursor into metallic Au. This second heat treatment promoted framework condensation, slightly thickening the pore walls in the SBA-11 structure. Such framework condensation is common in mesoporous silica structures that are produced using the TEOS precursor [28,29]. Additionally, the relative decrease in this peak intensity for both samples after the introduction of Au clusters in relation to that for pristine SBA-11 (the height differences of the peak labeled C for both samples are always smaller than that of D in Figure 1a,b, indicating that some of the pores are occupied, in this case with metallic Au nanoclusters). This was also confirmed by the decrease in the surface areas of both Au–SBA-11 samples discussed in Section 2.5. Moreover, the SBA-11 structure maintained the long-range order of pores. This suggests that pores are stacked in a repeated fashion throughout the material, as indicated by the presence of the diffraction peaks at 211, 300, 310, and 331 [30], and appeared intact after the introduction of the Au nanoclusters, as confirmed by the TEM images in Figure 3c and Figure 4f.

### 2.2. Wide-Angle XRD

**Figure 2:**
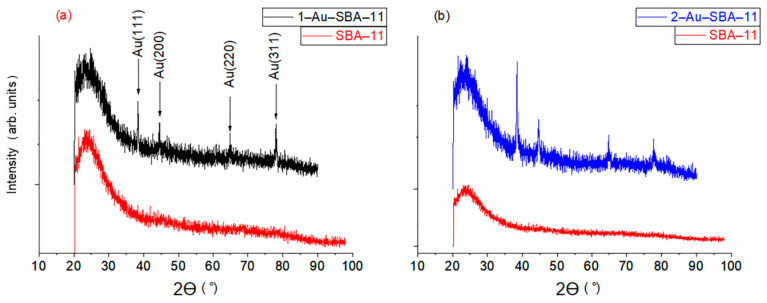
Wide-angle XRD for samples (**a**) 1–Au–SBA-11 and (**b**) 2–Au–SBA-11 in comparison with pristine SBA-11.

The wide-angle XRD patterns of 1–Au–SBA-11 (a) and 2–Au–SBA-11 (b) are compared with that of unloaded SBA-11 (red) in Figure 2. The broad Bragg peak at 2θ = 20–35°, which is present for all the SBA-11 samples, indicates that SBA-11 is amorphous silica. The other four diffraction peaks appear at 2θ = 30–80°, as indicated by the arrows, and correspond to crystalline metallic Au [3,9]. This confirms the formation of Au nanoclusters in SBA-11. Furthermore, these peaks were higher in intensity and broader for 2–Au–SBA-11 than for 1–Au–SBA-11. This indicates that the Au crystals were larger in 2–Au–SBA-11 than in 1–Au–SBA-11, as confirmed by the HRTEM images (vide infra).

### 2.3. HRTEM and EDX

#### 2.3.1. 1–Au–SBA-11

**Figure 3:**
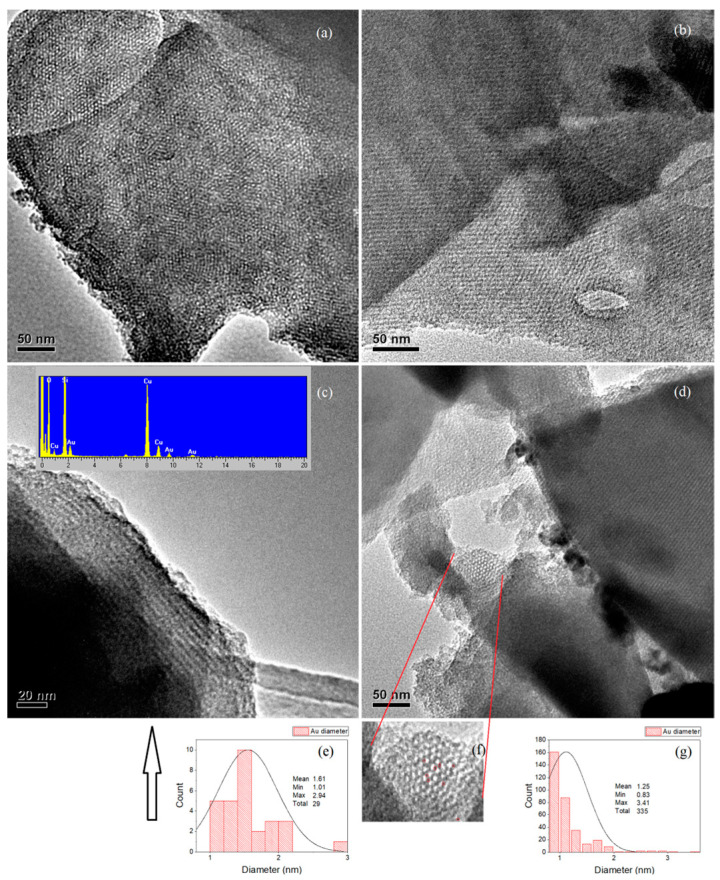
(**a**–**d**) HRTEM images of 1–Au–SBA-11; (**e**,**g**) corresponding Au cluster size distributions; (**f**) is a magnified section of image (**d**).

The HRTEM images of 1–Au–SBA-11 are shown in Figure 3. The image in Figure 3a reveals the mesoporous arrangement of SBA-11, showing a top view of the pore openings. This also confirms that the entire sample is porous, considering that SBA-11 pores have a cubic pore structure that is interconnected perpendicularly. This makes the pore arrangement and clusters accessible from multiple directions. Figure 3b presents another section of the sample, showing the long-range order of pores, which appear as diagonal parallel lines. The long-range order of pores is also visible in Figure 3c,d. Figure 3c shows another section of the sample at high magnification, revealing that the Au clusters (appearing darker) are embedded within the walls adjacent to the pores. The corresponding EDX spectrum in the inset of Figure 3c clearly shows the presence of all compositional elements in the sample: the K-edges of Si and O representing the SBA-11 silica structure, and the L-edge of Au representing the Au nanoclusters. Cu is the mesh on which the sample was mounted during TEM. The average Au content determined via EDX was 0.5 wt.%. Figure 3d, as well as Figures S1 and S2 in the Supplementary Information (SI), contain two main parts: the right side shows the clear array stacking of pores, with long-range order, in the silica host. The other section, which is trapped and contained within the two red lines, is the top view of the pore openings that was magnified in Figure 3f and Figure S2 in the SI. It clearly shows the locations of some of the Au clusters (outlined in red circles) and reveals that the Au clusters are circular. These clusters have an average diameter of 1.6 nm, as shown in the size distribution histogram in Figure 3e, with more details in Figure S4 in the SI. The sizes of the Au clusters are presented in the histogram (Figure 3g), which corresponds to Figure 3b. The Au cluster size distribution is extremely narrow (0.8–3.4 nm), which was also confirmed by the UV-vis spectra (Section 2.4). The average Au cluster size is 1.3 nm from 335 selected particles. More details of the chosen area and Au particles are presented in Figures S3 and S4 in the SI. Both histograms in Figure 3e,g reveal that the size of most Au clusters (having a higher count) falls between 1 and 2 nm. This indicates that converting the Au precursor to metallic Au clusters at 0–20 °C restricts their growth owing to pore/wall confinement during the second calcination step. During heat treatment, the Au clusters tended to transfer from the pores (nucleation sites) and diffuse into the silica walls, as can be clearly observed in Figure 3f and most parts of Figure 3a. This diffusion prevents Au clusters from leaching out of the matrix, which is beneficial for catalysis and drug delivery. Moreover, these small Au clusters were well distributed throughout the silica host, as clearly shown in Figure 3a,b and Figures S1–S4 in the SI. The Au clusters appear as darker spots owing to their higher density than that of silica.

#### 2.3.2. 2–Au–SBA-11

**Figure 4:**
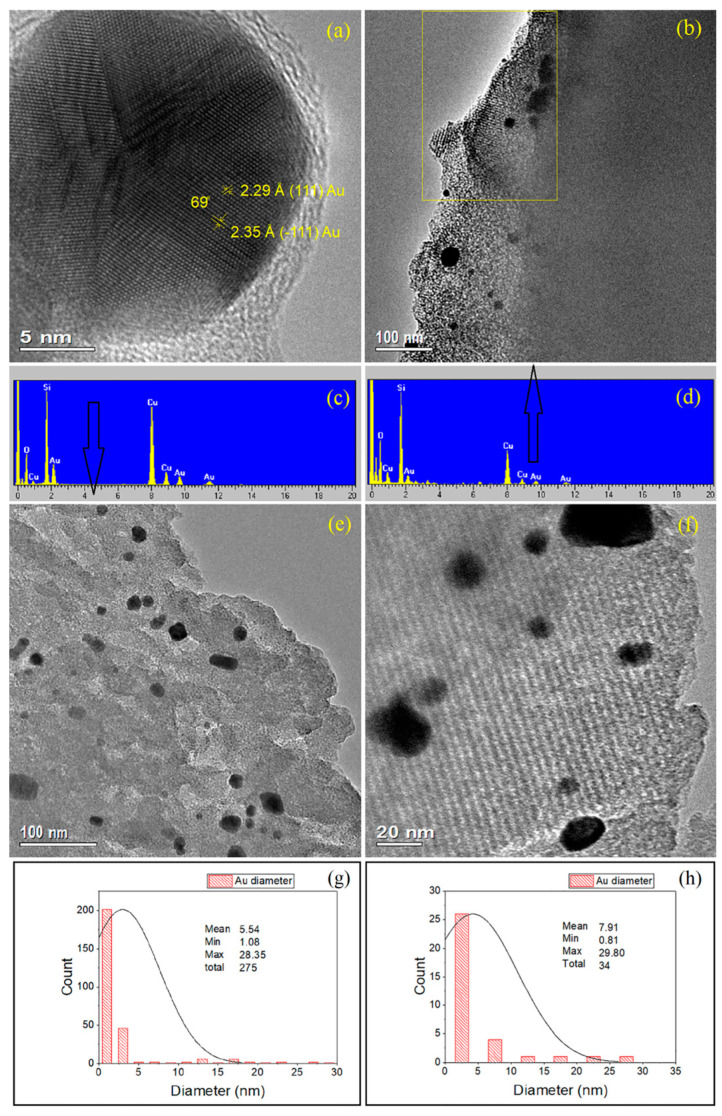
(**a**–**f**) HRTEM images, (**c**,**d**) EDX spectra, and (**g**,**h**) corresponding Au cluster sizes of 2–Au–SBA-11.

HRTEM (Figure 4) was conducted on 2–Au–SBA-11 to examine it in greater detail. Figure 4a shows a high-magnification image of a large terminal Au cluster (20 nm) embedded in 2–Au–SBA-11. The electronic diffraction of this cluster reveals diffraction planes (111) and (−111) of metallic Au. Figure 4b shows that the sample maintains a long-range order of mesopores, highlighted by the yellow rectangle. Additionally, there are two distinct sets of large and small Au clusters: the large clusters are randomly distributed across the sample, whereas the small clusters are confined within the pore wall arrangements (darker spots in all images). Figure 4c,d show the EDX spectra corresponding to Figure 4b,e, respectively. EDX shows the K- and L-edges of Si, O, and Au in the sample, confirming the silica composition of SBA-11 and the embedded Au clusters. The average Au content acquired by EDX was 0.7 wt.%. Figure 4e shows the HRTEM image of another section of the sample and confirms the presence of two types of Au clusters (large and small). The large clusters in Figure 4e are not uniform in size or shape. This was confirmed by UV-vis spectroscopy (vide infra; Section 2.4). Figure 4f shows an HRTEM image of a different section of the sample, which clearly reveals the long-range order of the pores of SBA-11, which appear as stacks of diagonal lines, as shown before in the low-angle XRD sections in Figure 1a,b, and the two types of Au clusters (large and small) embedded in the sample. Figure 4g,h are histograms of the Au cluster size distributions related to Figure 4e,f, respectively. These histograms confirm the presence of various cluster sizes, with the small Au clusters (1–2 nm) being the most abundant, accounting for 240 out of 275 clusters (Figure 4g). The large Au clusters range in size from 5 to 30 nm. More details about the selected Au particle sizes and locations within the images that were used in the histogram counts are provided in Figures S5 and S6 in the SI. The interpretation is that the emergence of two distinct sets of clusters occurred during the reduction heat treatment of the Au precursor during the second calcination step, at 400 °C. When the Au precursor was located inside the pores, it was transformed to small Au clusters of 1–2 nm in size by the pore and wall restrictions, as has been discussed previously in relation to Figure 3f. Conversely, when the Au precursor initially existed outside the pores or migrated through the porous structure during heat treatment (the second calcination step), large clusters (5–30 nm) were formed upon sintering and aggregation, without the pore and wall restrictions.

### 2.4. UV-Vis Spectra

**Figure 5:**
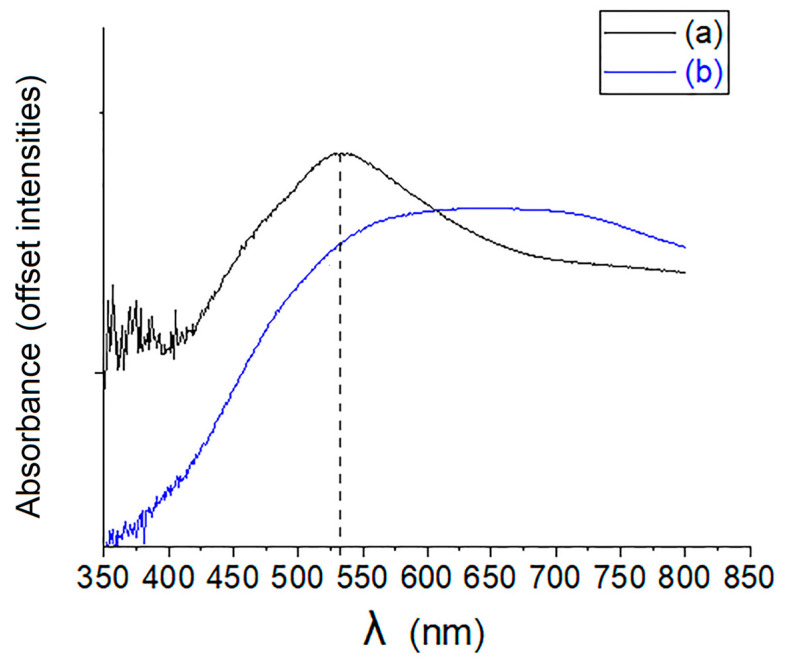
UV-vis spectra of (a) 1–Au–SBA-11 (black) and (b) 2–Au–SBA-11 (blue).

The UV-vis spectra for the two materials, 1–Au–SBA-11 and 2–Au–SBA-11, are shown in Figure 5. The spectrum of 2–Au–SBA-11 (blue) is very broad, extending from 425 to 800 nm. This indicates that the Au clusters within the silica matrix have a wide range of shapes and sizes, causing a redshift in the Au surface plasmon resonance peak [7,31], which agrees with the HRTEM images in Figure 4b,e,f. In contrast, 1–Au–SBA-11 has a narrow spectrum (black). This indicates narrow Au cluster sizes and shapes, consistent with the HRTEM images in Figure 3a–d. Furthermore, the Au surface plasmon resonance peak maximum at 530 nm for 1–Au–SBA-11 represents spherical and semispherical clusters [32], as confirmed by the HRTEM image in Figure 3f, instead of the commonly reported peak at 570 nm [9]. This blueshift in the Au plasmonic peak indicates that the Au clusters in 1–Au–SBA-11 are small [1,3] (1–2 nm) [33,34,35], as confirmed by the histogram cluster size distribution in Figure 3e,g. However, these differences in the plasmonic resonances of the samples may be beneficial in certain applications, as discussed in the Introduction. For example, for optical excitation devices that require a specific wavelength range that falls within 430–600 nm, 1–Au–SBA-11 could be employed, and if the requirements were for a larger range, then 2–Au–SBA-11 could be used. Differences in the shapes and sizes of Au clusters are also advantageous for delivery systems. Samples 1– and 2–Au–SBA-11 were used as photocatalysts to degrade M.B. under sunlight, demonstrating one of their practical applications.

### 2.5. N_2_ Sorption Analysis

**Figure 6:**
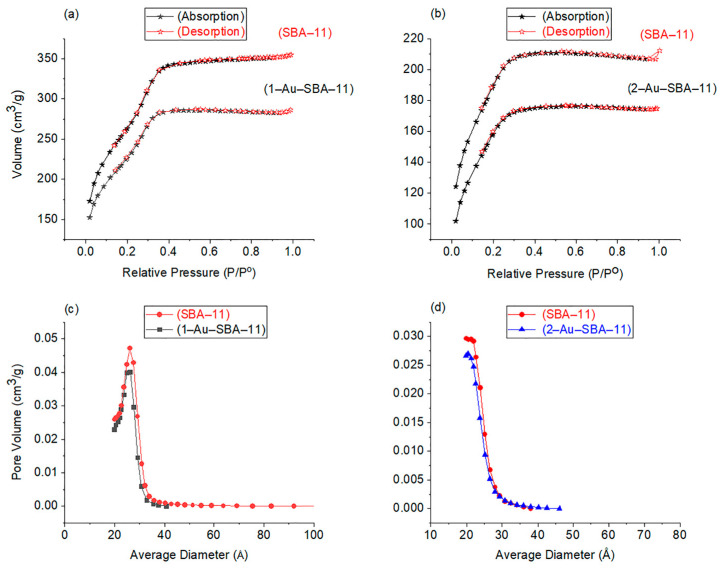
Nitrogen adsorption–desorption isotherms of (**a**) 1–Au–SBA-11 and (**b**) 2–Au–SBA-11. Pore size distributions of (**c**) 1–Au–SBA-11 and (**d**) 2–Au–SBA-11. All data are compared with those of pristine SBA-11.

N_2_ sorption analysis was conducted to further study the effect of the impregnation of Au nanoclusters within the SBA-11 pores (Figure 6). The isotherms of 1–Au–SBA-11 and 2–Au–SBA-11 are presented in Figure 6a,b, respectively, alongside that of the corresponding original unimpregnated SBA-11. Both samples produced the typical IVb isotherms for mesoporous materials [36]. No hysteresis was observed during the desorption cycle, indicating that the pore diameters were smaller than the critical size of 4 nm and uniformly shaped [36]. The shapes of the isotherms produced by 1–Au–SBA-11 and 2–Au–SBA-11 were similar to those of their original SBA-11 host, though with lower volume. This decline in volume was caused by some of the Au nanoclusters that partially occupied the pores, confirming that the impregnation process was successful, which produced lower intensities (pore volumes) in both graphs for both samples in Figure 6c,d. This volume reduction resulted in a decrease in the total surface area of both samples. The BET method was used to determine the surface areas, which were 940 and 675 m^2^g^−1^ for the original pristine SBA-11 sample and then decreased to 807 m^2^g^−1^ for 1–Au–SBA-11 and 567 m^2^g^−1^ for 2–Au–SBA-11, respectively. The surface area reduction after Au introduction was approximately 15.5% for both samples. The average pore diameters of 1–Au–SBA-11 and 2–Au–SBA-11 are compared with those of pristine SBA-11 in Figure 6c,d, respectively. The shifts in the 1–Au–SBA-11 and 2–Au–SBA-11 curves toward smaller diameters represent a slight shrinkage of ~0.1 nm in the average pore size relative to that of their SBA-11 hosts. These shifts also indicate that some Au clusters were occupying pores. The BJH method yielded an average pore size of 2.2 nm for 1–Au–SBA-11 and 2.0 nm for 2–Au–SBA-11.

### 2.6. XPS

**Figure 7:**
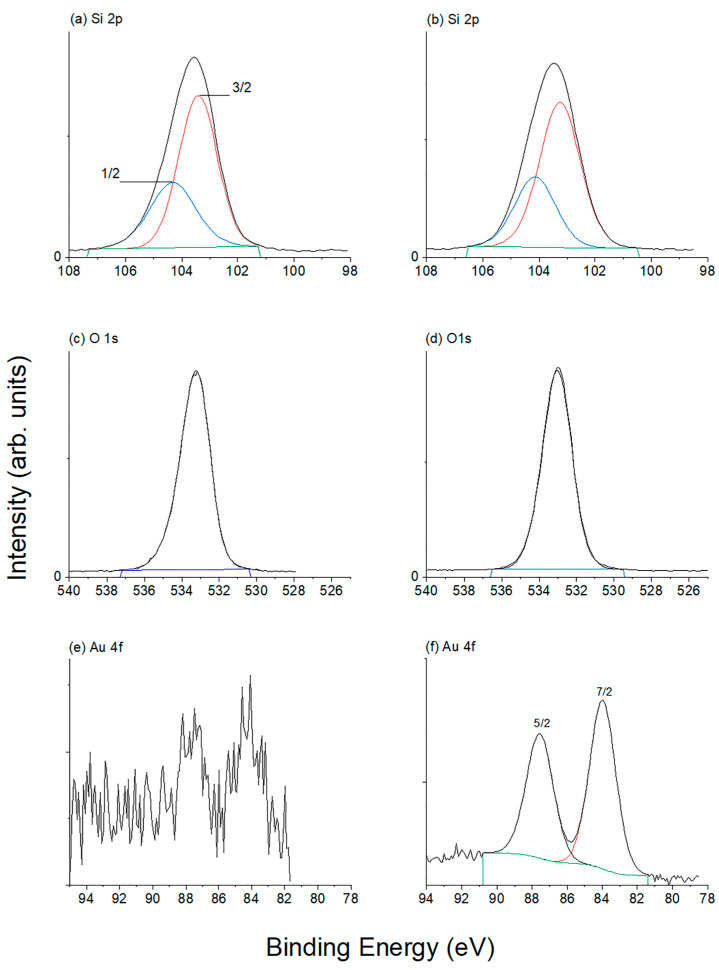
XP spectra of (**a**,**c**,**e**) 1–Au–SBA-11 and (**b**,**d**,**f**) 2–Au–SBA-11.

The oxidation states of Si, O, and Au in 1–Au–SBA-11 and 2–Au–SBA-11 were investigated through XPS. The results are presented in Figure 7. First, the prominent Si XPS peaks are similar for both samples in Figure 7a,b. The deconvolution of the main Si peak reveals two peaks at 103.4 [37] and 104.3 eV, corresponding to Si^4+^ 2p_3/2_ and 2p_1/2_ orbital splitting [38], respectively, and representing the formation of Si–O bonds within the SiO_2_ framework in both samples [24,39,40]. Second, the symmetrical O1s XPS peaks of 1–Au–SBA-11 and 2–Au–SBA-11, which are demonstrated in Figure 7c,d, respectively, at 533.3 eV [41] correspond to the framework O_2_ atoms in SiO_2_ [24,39,40]. Finally, two Au XPS peaks at 83.4 and 87.5 eV attributed to Au 4f_7/2_ and Au 4f_5/2_, having peak intensities in a 4:3 ratio [32], correspond to 4f orbital splitting and indicate the existence of metallic Au in both samples [7,9,32,41]: 1–Au–SBA-11 in Figure 7e and 2–Au–SBA-11 in Figure 7f. Full XPS scans of both materials, presented in Figure S7 in the SI, show no foreign elements.

### 2.7. Photocatalytic Activity of Au–SBA-11

**Figure 8:**
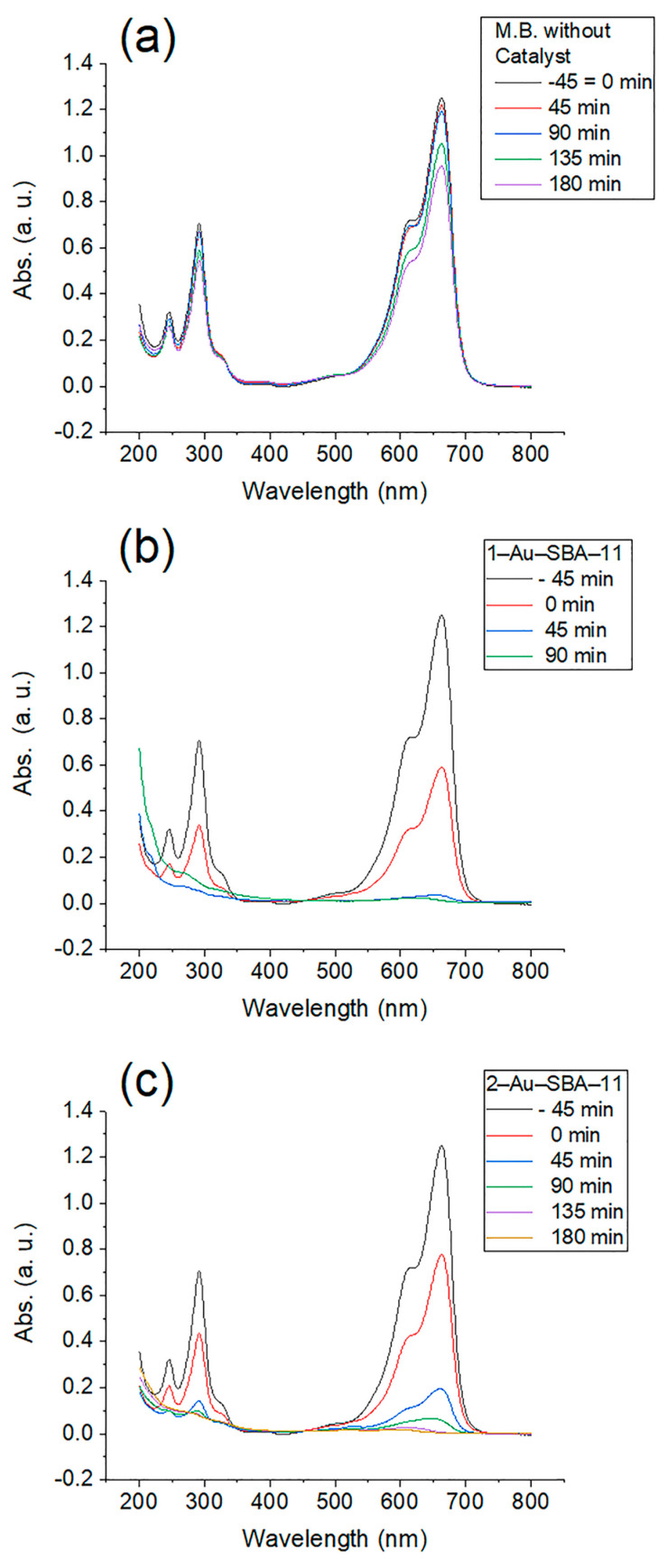
UV-vis absorption spectra of M.B. solution exposed to sun light (**a**) without catalyst, (**b**) with 1–Au–SBA-11, and (**c**) with 2–Au–SBA-11.

Figure 8 presents the UV-vis spectra of the solutions collected from the photocatalytic reaction at 45 min intervals under dark conditions (no sunlight) and upon direct sunlight exposure. An apparent change in the intensity of the main peak at 664 nm is observed compared with the intensity produced by the absorption of the starting concentration of 5 ppm, which is 1.22 in all spectra individually. Figure 8a presents the UV-vis spectrum of the M.B. solution without any catalyst. The maximum intensity decrease occurred after 180 min of sunlight exposure, calculated as 23% of the initial peak height. This calculation was based on Equation (1) and used to calculate the M.B. degradation percentage for all samples.

M.B. degradation % = (1 − *C*/*C*_0_) × 100 (1)

Here, *C* is the concentration of M.B. at any given time and *C*_0_ is the initial M.B. concentration. Figure 8b represents the spectra of M.B. in the presence of 1–Au–SBA-1. The first intensity belongs to the initial M.B. concentration, and the second one represents the sorption ability of 1–Au–SBA-1 under dark conditions for 45 min (hence the negative sign is assigned to it in the figure legend) before sunlight exposure that was monitored starting at 0 min. At the third reading, after 45 min of sun exposure, the peak height dropped significantly. Finally, after 90 min of direct sunlight exposure, the M.B. peak nearly flattened. Figure 8c represents the spectra of M.B. in the presence of 2–Au–SBA-11. M.B. degradation in the presence of 2–Au–SBA-11 followed the same scenario as that of 1–Au–SBA-11, but took 180 min instead of 90 min to thoroughly degrade in the solution.

**Figure 9:**
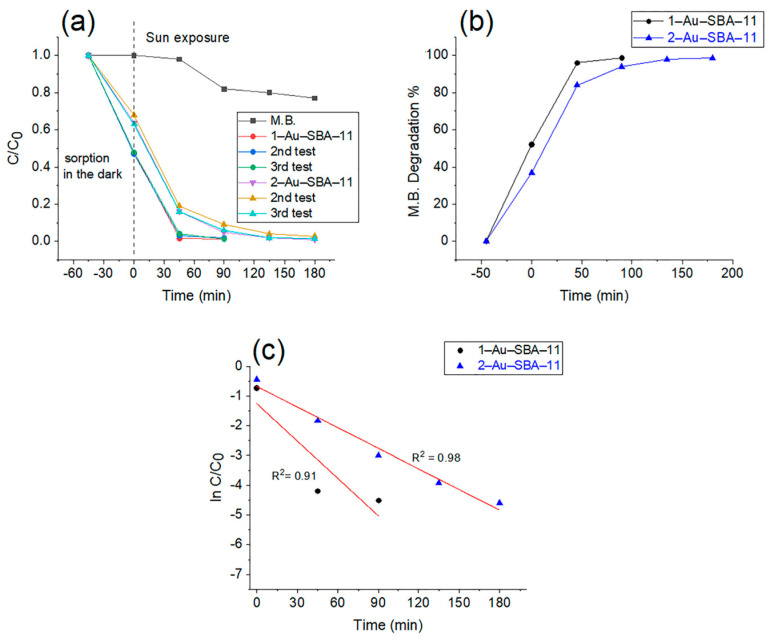
(**a**) Photocatalytic behavior of M.B. solution. (**b**) M.B. degradation percentage. (**c**) First-order plot of kinetic M.B. degradation over 1–Au–SBA-11 and 2–Au–SBA-11.

Figure 9a illustrates the change in the concentration of the M.B. solution over time in relation to the initial concentration, with and without the materials. The first section of this graph, from −45 to 0 min, indicated by the dashed line, represents the dark conditions before the materials were exposed to sunlight. This illustrates the sorption ability of each material, which was higher for 1–Au–SBA-11 than for 2–Au–SBA-11. This difference in the sorption ability of the materials corresponds to the differences in the surface area between the samples, with 1–Au–SBA-11 having a larger surface area than 2–Au–SBA-11, as discussed in Section 2.5. The second section, from 0 to 180 min, after the dashed line, represents the change in the M.B. solution concentration due to exposure to direct sunlight. First, the black line with solid black squares represents the M.B. solution without any catalyst. The value of M.B. fraction concentration *C/C*_0_ decreased to 0.77 from the initial concentration of 1.0, which represents a total loss of 23% of the initial concentration after 180 min of sunlight exposure. The second group of three lines, which contain solid circles (red, blue, and green), represents the degradation of M.B. by sunlight due to 1–Au–SBA-11. These three lines, representing three different tests, are nearly superimposed. The maximum standard deviation values for these tests were 0.01253 for 1–Au–SBA-11 and 0.02646 for 2–Au–SBA-11, as shown in Figure S8 in the SI. This indicates that the largest differences among the three tests deviated minimally from the mean, providing high confidence in the results. The three lines show that the presence of 1–Au–SBA-11 increased the degradation of the M.B. solution to reach a *C/C_0_* value of 0.01 of its initial concentration after 90 min of sunlight exposure, decomposing M.B. in the solution. The third set of lines (lilac, brown, and turquoise, marked with triangles) represents three tests of M.B. solution with 2–Au–SBA-11. These lines in Figure 9a show that 0.99 of the M.B. solution is decomposed after being exposed to sunlight for 180 min to reach a *C/C_0_* value of 0.01 of its initial concentration. Figure 9b shows the percentage loss of M.B. in solution in the presence of each material upon sunlight exposure. Sample 1–Au–SBA-11 is indicated by the black line with solid circles. The graph clearly shows that 99% of the M.B. solution decomposes after 90 min of sunlight exposure in the presence of 1–Au–SBA-11, whereas 2–Au–SBA-11 requires 180 min of sunlight exposure to degrade 99% of the M.B. Figure 9a,b clearly show that 2–Au–SBA-11 took twice as long as 1–Au–SBA-11 to decompose M.B. in the presence of sunlight under identical reaction conditions. Figure 9c shows the reaction kinetics of these photodegradation reactions [42], which follow the pseudo-first-order kinetics described by Equation (2).

*ln*(*C*/*C*_0_) = *k t*, (2)

Here, *C* is the concentration of M.B. at any given time *t*, *C*_0_ is the initial M.B. concentration, and *k* is the first-order rate constant; *k* is determined from the slope of *ln(C/C*_0_*)* versus time in Figure 9c. The obtained *k* value is (0.04185 ± 0.00905) min^−1^ with a regression coefficient (R^2^) of 0.91 for 1–Au–SBA-11 and (0.0231 ± 0.00177) min^−1^ with R^2^ of 0.98 for 2–Au–SBA-11. Both R^2^ values indicate high confidence for the linear fitted red lines. The *k* value of 1–Au–SBA-11 is twice that of 2–Au–SBA-11, indicating a higher reaction rate. This is also confirmed by the time required to degrade M.B. when 1–Au–SBA-11 is used: 90 min compared to 180 min for 2–Au–SBA-11 (Figure 9b). The proposed photocatalytic mechanism involves sunlight photons exciting electrons in the Au nanoclusters, resulting in radical formation in the solution. Additionally, the Au nanoclusters act as mediators, transferring electrons from M.B. to oxygen. These oxygen molecules accept the electrons to become O_2_^•^ radicals and then form more radicals, such as HO^•^ and HO_2_^•^, in the solutions [43]. These radicals attack M.B. molecules and decompose them into smaller fragments [43]. Moreover, the large surface area of 1–Au–SBA-11 enhances M.B. adsorption, enabling more M.B. molecules to interact with additional Au clusters, thereby promoting electron transfer.

**Figure 10:**
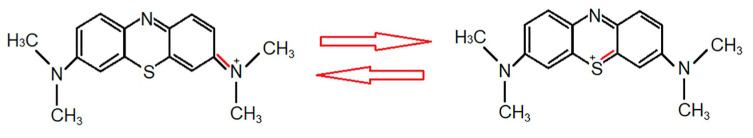
Schematic representation of resonance in a single M.B. molecule.

The structure of M.B. molecules contains many conjugated double bonds and positive centers. These positive centers resonate with the double bonds of the aromatic rings, as shown in Figure 10, and are electrostatically attracted to the excited electrons of the Au clusters. The double bonds of M.B. (which are negatively charged) are also attracted to the positive surfaces of the Au clusters [43,44] as a result of the plasmonic effect. Then, after M.B. comes into contact with the Au, the electrons transfer from M.B. to oxygen molecules in the reaction solution, transforming them into O_2_^•^ free radicals. These O_2_^•^ are very active, and they attack the M.B. molecules and other water molecules to create more radical species, which are also highly active. These newly generated radicals in turn also attack M.B. and other water molecules, creating chain reactions until the M.B. is completely degraded.

## 3. Materials and Methods

### 3.1. Synthesis of Mesoporous Silica (SBA-11)

All chemicals were purchased from Sigma-Aldrich, St. Louis, MI, USA, and used without further purification. First, 4.0 g of Brij 56 (a surfactant; C_36_H_74_O_11_) was dissolved by stirring it into a mixture of 20 mL deionized water and 80 mL 2 M HCl at 40 °C and then cooled to room temperature. Tetraethoxysilane (TEOS, 8.8 g) was then added, and the mixture was stirred overnight (12–16 h). The mixture was then filtered and washed with deionized water and ethanol. The resulting powder was dried at room temperature for 12–16 h. Consequently, the as-synthesized powder was calcined in static air at 500 °C for 6 h at 1.1 °C min^−1^ and then cooled to room temperature at the same rate [30].

### 3.2. Synthesis of Nanosized Au in Mesoporous Silica

Mesoporous silica (1.0 g) was heated at 150 °C for 2 h under N_2_ flow, and 0.01 g of KAuCl_4_ was dissolved in 10 mL of methanol at 0 °C. The dried mesoporous silica was stirred for 30 min into the Au solution at 0 °C, during which the round-bottomed flask containing the mixture was covered with aluminum foil. Next, 2% H_2_ in N_2_ (Refrigeration and Oxygen Co., Ltd., Safat, Kuwait) was introduced to the mix at 20 °C and bubbling was continued for 30 min. The second batch was treated the same way, except that H_2_ gas was not introduced to the mixture solution of the Au precursor and silica. Both samples were heated at 40 °C until dry, washed twice with ethanol, filtered, and air-dried overnight. Both batches were calcined at 400 °C for 1 h under 2% H_2_ in a 98% N_2_ gas mixture, and then in static (ambient) air for 2 h at a rate of 3 °C min^−1^. Finally, the samples were cooled to room temperature at the same rate of 3 °C min^−1^. The first batch was labeled 1–Au–SBA-11, and the second batch was labeled 2–Au–SBA-11.

### 3.3. Photocatalytic Activity of Au–SBA-11

The photocatalytic activity of the Au–SBA-11 samples was analyzed by stirring 35 mg of each Au–SBA-11 material into 35 mL methylene blue (M.B., Merck, Darmstadt, Germany) solution (5 ppm) in deionized water in a 50 mL Pyrex round-bottomed flask (Sigma-Aldrich). The flask was kept in the dark for 45 min, followed by exposure to direct sunlight for 180 min from 11:00 a.m. to 2:00 p.m., when the average solar power was 970 W/m^2^. The reaction was cooled in ambient air at 18 °C ± 1 °C in February 2025, and the solution temperature was 28 ± 1 °C. Aliquots of 3.5 mL were withdrawn at 45 min intervals using a reusable 5 mL syringe and then analyzed using a Cary 5000 ultraviolet-visible–near infrared (UV-vis-NIR) spectrophotometer (Agilent, Santa Clara, CA, USA) to monitor the change in the maximum intensity of M.B. at 664 nm. One sample of M.B. was used without a catalyst during these tests as a control sample. The photocatalytic tests were repeated three times on three different days.

### 3.4. Characterization Methods

Powder X-ray diffraction (XRD) was conducted using a Bruker AXS D8 Advance diffractometer (Billerica, MA, USA) with a copper target (λ = 1.5418 Å). DIFFRACplus software (version 3.1) was used for analysis, and the following operational parameters were used: 40 kV, 40 mA, and a front slit window of size 0.1 mm for narrow-angle scans and 1.0 mm for wide-angle scans. The deflection plate was placed 0.5–1.0 mm above the sample for the low-angle scan in the continuous coupled 2*θ* scan/*θ* scan mode at 0.5 step/s. Nitrogen gas adsorption–desorption analysis was performed using a Micrometrics Tristar analyzer. The surface areas were determined using the Brunauer–Emmett–Teller (BET) method, whereas the average pore sizes were obtained through Barrett–Joyner–Halenda (BJH) adsorption analysis. High-resolution transmission electron microscopy (HRTEM) was performed using a JEOL JEM-3011 electron microscope (Akishima, Japan) operating at 250 kV. The images were recorded using a Gatan 794 charge-coupled device camera (Pleasanton, CA, USA). Gatan Digital Micrograph^®^ software (https://www.gatan.com/products/tem-analysis/gatan-microscopy-suite-software) was used to analyze the transmission electron microscopy (TEM) images. ImageJ software version 1.54 was used to measure the pores and cluster sizes in the TEM images. The TEM instrument JEOL JEM-3010 electron microscope (Akishima, Japan) was equipped with a PGT prism Si (Li) energy-dispersive X-ray spectroscopy (EDX) detector and an Avalon 2000 analyzer (Carlsbad, CA, USA), which were used for determining the chemical composition of the samples. EDX was conducted in the TEM mode using a 7 nm beam diameter for an acquisition time of 1 min. X-ray photoelectron spectroscopy (XPS) was performed using a Thermo ESCALAB 250Xi spectrometer (Waltham, MA, USA) with a monochromator and an Al-Kα radiation source (1486.6 eV). The spectra were recorded and processed utilizing an Avantage data system. The analyses were carried out using the following parameters: analysis chamber pressure of 10^–9^ Torr, step size of 0.1 eV, dwell time of 100 ms, and pass energy of 20 eV. All binding energy values were determined with respect to the C1s line (284.6 eV) originating from adventitious carbon. A flood gun was used in the standard charge compensation mode to neutralize the charge buildup on the surface of the insulating layer. UV-vis spectroscopy was conducted using a Cary 5000 UV-vis–NIR spectrophotometer (version 1.12) with the following settings: abs mode 200–800 nm, scan rate 600.000 nm min^−1^, data interval 1.000 nm, full slit height, double beam mode, signal-to-noise mode off, and baseline correction on. The powder samples were pressed into a disk shape without dilution, and an SBA-11 disk was used as the reference sample (zero baseline) for the UV-vis spectroscopy. Graphs were constructed using Origin software version 95E, including the linear fit.

## 4. Conclusions

This paper presented a facile method for controlling the size of Au clusters within a silica host. Introducing a reducing gas to the Au precursor at low temperatures was found to be important to maintaining small Au clusters within the porous structure. However, introducing a reducing gas during heat treatment at elevated temperatures produced two sets of Au clusters in the final product: small clusters within and large clusters outside the pores. These larger clusters formed through the aggregation of the Au precursor that was initially outside the pores or migrated during heat treatment. Moreover, the predominance of small Au clusters in 1–Au–SBA-11 doubled the M.B. degradation rate in relation to that of 2–Au–SBA-11.

## Data Availability

The data that support the findings of this study are found in the Supplementary Materials. Additional data are available from the corresponding author upon reasonable request.

## Supplementary Materials

The following supporting information can be downloaded at https://www.mdpi.com/article/10.3390/molecules30092035/s1.

## Abbreviations

The following abbreviations are used in this manuscript:

XRD	X-ray diffraction
EDX	energy-dispersive X-ray spectroscopy
HRTEM	high-resolution transmission electron microscopy
UV-vis	ultraviolet-visible
XPS	X-ray photoelectron spectroscopy
GI	gastrointestinal
Brij 56	C_36_H_74_O_11_, also called C_16_EO_10_, polyethylene glycol hexadecyl ether, or polyoxyethylene (10) cetyl ether
TEOS	tetraethoxysilane
M.B.	methylene blue
Arb	Arbitrary
