# Supplementary material for: Simple Method for Controlling Gold Nanocluster Size in Mesoporous Silica: SBA-11
Source: Molecules. 2025 May 2;30(9):2035. doi: 10.3390/molecules30092035 (PMC12073877; doi:10.3390/molecules30092035)
Supplement: Supplementary file 1 [file molecules-30-02035-s001.zip › molecules-3559464-supplementary.pdf]

Supplementary Material

Simple Method for Controlling Gold Nanocluster Size in Mesoporous Silica: SBA–11

Tariq Aqeel [1,] * and Ali Bumajdad [2]

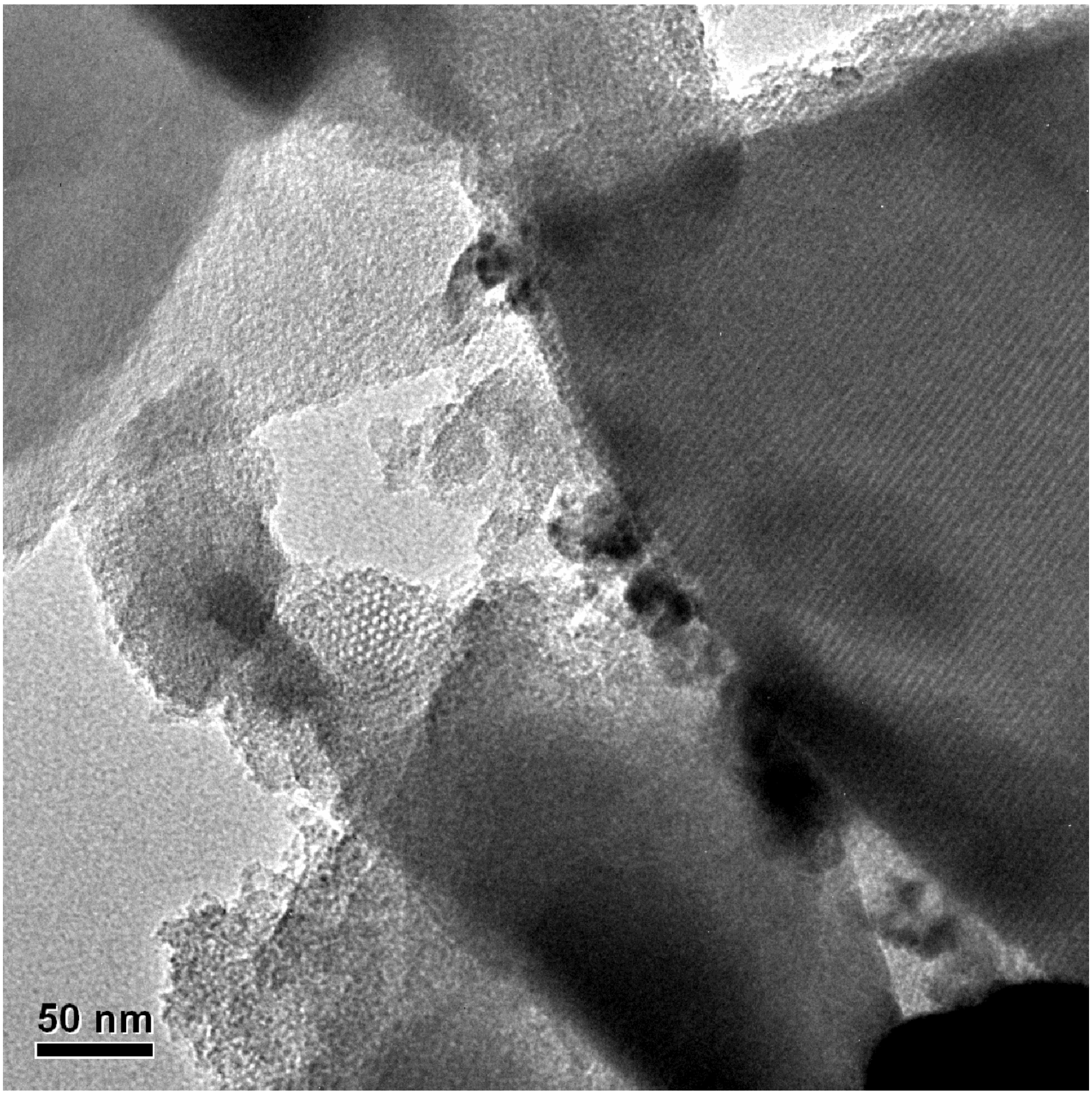

Figure S1. Original HRTEM image of 1–Au–SBA–11, without highlighting, which is presented in Fig. 3(d).

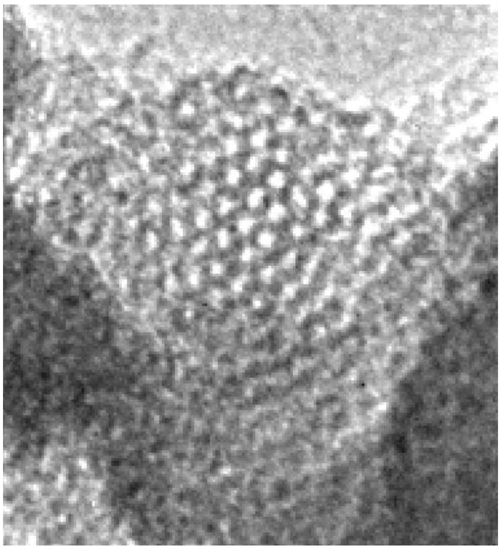

Figure S2. Section of S1 that is magnified to show the Au clusters that are imbedded in the pore walls of SBA–11 without highlighting, shown in Figure 3(f) in the text.

The bright-field HRTEM image in Figure S2 is a highly magnified image of Figure S1, and 3(d), which is presented in the main manuscript, clearly shows that some of the exposed pores have sizes of 2.0 to 2.2 nm (measured by ImageJ). There are a few dark spots in the bright-field TEM image (Figure S2). These indicate the Au clusters (confirmed by the EDX and XPS), because the atomic weight of Au is 197 g/mol which is more than the 28 g/mol for Si, and therefore Au-regions should appear darker than Si in bright-field TEM images.

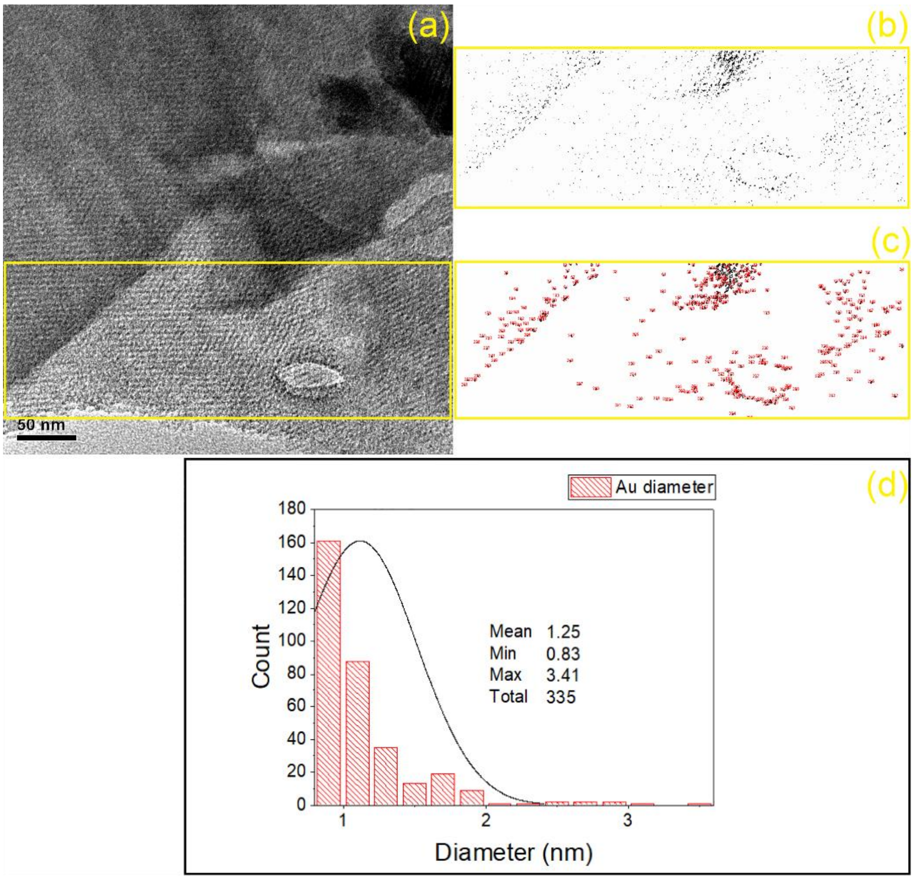

Figure S3. (a) Represent the selected section HRTEM image of 1–Au–SBA–11 Figure 3(b) in the text, (b) is the adjusted contrast of (a), (c) the chosen particles numbered from (b), and (d) the produced particles size distribution.

Figure S3 (a) represents the selected area of image 3(b) in the text highlighted in yellow. While S3 (b) shows the Au clusters clearer after adjusting the contrast. S3 (c) showing the chosen Au particles and their labels of the selected area. S3 (d) is the produced histogram of the selected Au clusters. These selected Au clusters and their parameters are listed in Table S1.

Table S1 showing the numbered Au particles and their parameters

d (nm)	r	r^2	Max	Min	Mean	Area	Label
1.016168	0.508084	0.258149	255	255	255	0.811	1
1.606504	0.803252	0.645214	255	255	255	2.027	2
0.829953	0.414977	0.172206	255	255	255	0.541	3
2.874378	1.437189	2.065513	255	255	255	6.489	4
1.900909	0.950454	0.903363	255	255	255	2.838	5
2.234077	1.117038	1.247775	255	255	255	3.92	6
0.927745	0.463872	0.215177	255	255	255	0.676	7
1.659523	0.829762	0.688504	255	255	255	2.163	8
1.244291	0.622145	0.387065	255	255	255	1.216	9
1.244291	0.622145	0.387065	255	255	255	1.216	10
0.829953	0.414977	0.172206	255	255	255	0.541	11
0.927745	0.463872	0.215177	255	255	255	0.676	12

0.927745	0.463872	0.215177	255	255	255	0.676	13
0.829953	0.414977	0.172206	255	255	255	0.541	14
0.927745	0.463872	0.215177	255	255	255	0.676	15
1.606504	0.803252	0.645214	255	255	255	2.027	16
1.900909	0.950454	0.903363	255	255	255	2.838	17
0.927745	0.463872	0.215177	255	255	255	0.676	18
2.796469	1.398234	1.955059	255	255	255	6.142	19
1.375975	0.687988	0.473327	255	255	255	1.487	20
1.09749	0.548745	0.301121	255	255	255	0.946	21
0.927745	0.463872	0.215177	255	255	255	0.676	22
1.375975	0.687988	0.473327	255	255	255	1.487	23
1.016168	0.508084	0.258149	255	255	255	0.811	24
0.829953	0.414977	0.172206	255	255	255	0.541	25
2.782776	1.391388	1.935961	255	255	255	6.082	26
1.016168	0.508084	0.258149	255	255	255	0.811	27
1.09749	0.548745	0.301121	255	255	255	0.946	28
0.829953	0.414977	0.172206	255	255	255	0.541	29
1.173189	0.586594	0.344093	255	255	255	1.081	30
1.659523	0.829762	0.688504	255	255	255	2.163	31
1.016168	0.508084	0.258149	255	255	255	0.811	32
0.829953	0.414977	0.172206	255	255	255	0.541	33
1.016168	0.508084	0.258149	255	255	255	0.811	34
0.927745	0.463872	0.215177	255	255	255	0.676	35
1.09749	0.548745	0.301121	255	255	255	0.946	36
0.829953	0.414977	0.172206	255	255	255	0.541	37
0.829953	0.414977	0.172206	255	255	255	0.541	38
0.927745	0.463872	0.215177	255	255	255	0.676	39
1.552085	0.776043	0.602242	255	255	255	1.892	40
1.437078	0.718539	0.516299	255	255	255	1.622	41
0.927745	0.463872	0.215177	255	255	255	0.676	42
1.989598	0.994799	0.989625	255	255	255	3.109	43
1.09749	0.548745	0.301121	255	255	255	0.946	44
3.471302	1.735651	3.012485	255	255	255	9.464	45
0.927745	0.463872	0.215177	255	255	255	0.676	46
0.927745	0.463872	0.215177	255	255	255	0.676	47
0.829953	0.414977	0.172206	255	255	255	0.541	48
1.312029	0.656014	0.430355	255	255	255	1.352	49
1.606504	0.803252	0.645214	255	255	255	2.027	50
0.927745	0.463872	0.215177	255	255	255	0.676	51
1.495688	0.747844	0.55927	255	255	255	1.757	52
0.829953	0.414977	0.172206	255	255	255	0.541	53
0.829953	0.414977	0.172206	255	255	255	0.541	54
1.606504	0.803252	0.645214	255	255	255	2.027	55
1.016168	0.508084	0.258149	255	255	255	0.811	56
1.173189	0.586594	0.344093	255	255	255	1.081	57
1.016168	0.508084	0.258149	255	255	255	0.811	58
0.927745	0.463872	0.215177	255	255	255	0.676	59
1.375975	0.687988	0.473327	255	255	255	1.487	60
2.874157	1.437078	2.065195	255	255	255	6.488	61
1.016168	0.508084	0.258149	255	255	255	0.811	62

0.927745	0.463872	0.215177	255	255	255	0.676	63
1.173189	0.586594	0.344093	255	255	255	1.081	64
0.829953	0.414977	0.172206	255	255	255	0.541	65
0.829953	0.414977	0.172206	255	255	255	0.541	66
1.016168	0.508084	0.258149	255	255	255	0.811	67
1.09749	0.548745	0.301121	255	255	255	0.946	68
0.829953	0.414977	0.172206	255	255	255	0.541	69
0.927745	0.463872	0.215177	255	255	255	0.676	70
0.829953	0.414977	0.172206	255	255	255	0.541	71
0.829953	0.414977	0.172206	255	255	255	0.541	72
0.927745	0.463872	0.215177	255	255	255	0.676	73
0.829953	0.414977	0.172206	255	255	255	0.541	74
0.829953	0.414977	0.172206	255	255	255	0.541	75
1.375975	0.687988	0.473327	255	255	255	1.487	76
0.829953	0.414977	0.172206	255	255	255	0.541	77
1.173189	0.586594	0.344093	255	255	255	1.081	78
1.375975	0.687988	0.473327	255	255	255	1.487	79
2.523385	1.261692	1.591868	255	255	255	5.001	80
1.312029	0.656014	0.430355	255	255	255	1.352	81
0.829953	0.414977	0.172206	255	255	255	0.541	82
0.829953	0.414977	0.172206	255	255	255	0.541	83
1.606504	0.803252	0.645214	255	255	255	2.027	84
1.09749	0.548745	0.301121	255	255	255	0.946	85
1.437078	0.718539	0.516299	255	255	255	1.622	86
1.760054	0.880027	0.774448	255	255	255	2.433	87
1.173189	0.586594	0.344093	255	255	255	1.081	88
0.829953	0.414977	0.172206	255	255	255	0.541	89
0.829953	0.414977	0.172206	255	255	255	0.541	90
1.244291	0.622145	0.387065	255	255	255	1.216	91
0.829953	0.414977	0.172206	255	255	255	0.541	92
0.829953	0.414977	0.172206	255	255	255	0.541	93
1.09749	0.548745	0.301121	255	255	255	0.946	94
1.016168	0.508084	0.258149	255	255	255	0.811	95
1.016168	0.508084	0.258149	255	255	255	0.811	96
1.016168	0.508084	0.258149	255	255	255	0.811	97
0.829953	0.414977	0.172206	255	255	255	0.541	98
1.09749	0.548745	0.301121	255	255	255	0.946	99
1.855146	0.927573	0.860392	255	255	255	2.703	100
1.989598	0.994799	0.989625	255	255	255	3.109	101
0.829953	0.414977	0.172206	255	255	255	0.541	102
0.829953	0.414977	0.172206	255	255	255	0.541	103
2.489093	1.244546	1.548896	255	255	255	4.866	104
0.829953	0.414977	0.172206	255	255	255	0.541	105
1.173189	0.586594	0.344093	255	255	255	1.081	106
0.829953	0.414977	0.172206	255	255	255	0.541	107
1.016168	0.508084	0.258149	255	255	255	0.811	108
3.186548	1.593274	2.538521	255	255	255	7.975	109
0.829953	0.414977	0.172206	255	255	255	0.541	110
1.808225	0.904113	0.81742	255	255	255	2.568	111
1.09749	0.548745	0.301121	255	255	255	0.946	112

0.829953	0.414977	0.172206	255	255	255	0.541	113
0.829953	0.414977	0.172206	255	255	255	0.541	114
1.760054	0.880027	0.774448	255	255	255	2.433	115
1.244291	0.622145	0.387065	255	255	255	1.216	116
1.659523	0.829762	0.688504	255	255	255	2.163	117
1.495688	0.747844	0.55927	255	255	255	1.757	118
1.375975	0.687988	0.473327	255	255	255	1.487	119
1.312029	0.656014	0.430355	255	255	255	1.352	120
0.829953	0.414977	0.172206	255	255	255	0.541	121
1.312029	0.656014	0.430355	255	255	255	1.352	122
1.173189	0.586594	0.344093	255	255	255	1.081	123
0.829953	0.414977	0.172206	255	255	255	0.541	124
1.016168	0.508084	0.258149	255	255	255	0.811	125
0.927745	0.463872	0.215177	255	255	255	0.676	126
1.855146	0.927573	0.860392	255	255	255	2.703	127
0.829953	0.414977	0.172206	255	255	255	0.541	128
0.829953	0.414977	0.172206	255	255	255	0.541	129
1.495688	0.747844	0.55927	255	255	255	1.757	130
0.829953	0.414977	0.172206	255	255	255	0.541	131
0.927745	0.463872	0.215177	255	255	255	0.676	132
0.829953	0.414977	0.172206	255	255	255	0.541	133
0.829953	0.414977	0.172206	255	255	255	0.541	134
1.244291	0.622145	0.387065	255	255	255	1.216	135
1.710528	0.855264	0.731476	255	255	255	2.298	136
1.09749	0.548745	0.301121	255	255	255	0.946	137
1.09749	0.548745	0.301121	255	255	255	0.946	138
0.927745	0.463872	0.215177	255	255	255	0.676	139
0.829953	0.414977	0.172206	255	255	255	0.541	140
1.312029	0.656014	0.430355	255	255	255	1.352	141
1.016168	0.508084	0.258149	255	255	255	0.811	142
1.900909	0.950454	0.903363	255	255	255	2.838	143
0.829953	0.414977	0.172206	255	255	255	0.541	144
1.016168	0.508084	0.258149	255	255	255	0.811	145
0.829953	0.414977	0.172206	255	255	255	0.541	146
0.927745	0.463872	0.215177	255	255	255	0.676	147
0.829953	0.414977	0.172206	255	255	255	0.541	148
1.173189	0.586594	0.344093	255	255	255	1.081	149
0.829953	0.414977	0.172206	255	255	255	0.541	150
0.829953	0.414977	0.172206	255	255	255	0.541	151
1.173189	0.586594	0.344093	255	255	255	1.081	152
0.829953	0.414977	0.172206	255	255	255	0.541	153
1.312029	0.656014	0.430355	255	255	255	1.352	154
1.760054	0.880027	0.774448	255	255	255	2.433	155
1.855146	0.927573	0.860392	255	255	255	2.703	156
1.244291	0.622145	0.387065	255	255	255	1.216	157
0.927745	0.463872	0.215177	255	255	255	0.676	158
1.312029	0.656014	0.430355	255	255	255	1.352	159
0.829953	0.414977	0.172206	255	255	255	0.541	160
0.927745	0.463872	0.215177	255	255	255	0.676	161
0.829953	0.414977	0.172206	255	255	255	0.541	162

0.829953	0.414977	0.172206	255	255	255	0.541	163
0.927745	0.463872	0.215177	255	255	255	0.676	164
0.829953	0.414977	0.172206	255	255	255	0.541	165
1.016168	0.508084	0.258149	255	255	255	0.811	166
0.829953	0.414977	0.172206	255	255	255	0.541	167
1.375975	0.687988	0.473327	255	255	255	1.487	168
0.829953	0.414977	0.172206	255	255	255	0.541	169
1.606504	0.803252	0.645214	255	255	255	2.027	170
1.016168	0.508084	0.258149	255	255	255	0.811	171
0.927745	0.463872	0.215177	255	255	255	0.676	172
1.09749	0.548745	0.301121	255	255	255	0.946	173
1.173189	0.586594	0.344093	255	255	255	1.081	174
0.927745	0.463872	0.215177	255	255	255	0.676	175
1.09749	0.548745	0.301121	255	255	255	0.946	176
1.016168	0.508084	0.258149	255	255	255	0.811	177
1.659523	0.829762	0.688504	255	255	255	2.163	178
0.927745	0.463872	0.215177	255	255	255	0.676	179
0.927745	0.463872	0.215177	255	255	255	0.676	180
0.829953	0.414977	0.172206	255	255	255	0.541	181
0.829953	0.414977	0.172206	255	255	255	0.541	182
1.437078	0.718539	0.516299	255	255	255	1.622	183
0.829953	0.414977	0.172206	255	255	255	0.541	184
0.829953	0.414977	0.172206	255	255	255	0.541	185
0.829953	0.414977	0.172206	255	255	255	0.541	186
1.312029	0.656014	0.430355	255	255	255	1.352	187
1.016168	0.508084	0.258149	255	255	255	0.811	188
0.829953	0.414977	0.172206	255	255	255	0.541	189
1.016168	0.508084	0.258149	255	255	255	0.811	190
0.927745	0.463872	0.215177	255	255	255	0.676	191
1.016168	0.508084	0.258149	255	255	255	0.811	192
1.016168	0.508084	0.258149	255	255	255	0.811	193
1.173189	0.586594	0.344093	255	255	255	1.081	194
0.927745	0.463872	0.215177	255	255	255	0.676	195
0.829953	0.414977	0.172206	255	255	255	0.541	196
1.659523	0.829762	0.688504	255	255	255	2.163	197
0.927745	0.463872	0.215177	255	255	255	0.676	198
1.016168	0.508084	0.258149	255	255	255	0.811	199
0.829953	0.414977	0.172206	255	255	255	0.541	200
1.312029	0.656014	0.430355	255	255	255	1.352	201
0.927745	0.463872	0.215177	255	255	255	0.676	202
1.09749	0.548745	0.301121	255	255	255	0.946	203
1.710528	0.855264	0.731476	255	255	255	2.298	204
0.829953	0.414977	0.172206	255	255	255	0.541	205
0.927745	0.463872	0.215177	255	255	255	0.676	206
1.016168	0.508084	0.258149	255	255	255	0.811	207
1.173189	0.586594	0.344093	255	255	255	1.081	208
1.016168	0.508084	0.258149	255	255	255	0.811	209
1.09749	0.548745	0.301121	255	255	255	0.946	210
0.927745	0.463872	0.215177	255	255	255	0.676	211
0.829953	0.414977	0.172206	255	255	255	0.541	212

0.829953	0.414977	0.172206	255	255	255	0.541	213
0.829953	0.414977	0.172206	255	255	255	0.541	214
1.09749	0.548745	0.301121	255	255	255	0.946	215
0.829953	0.414977	0.172206	255	255	255	0.541	216
0.927745	0.463872	0.215177	255	255	255	0.676	217
1.016168	0.508084	0.258149	255	255	255	0.811	218
1.016168	0.508084	0.258149	255	255	255	0.811	219
0.829953	0.414977	0.172206	255	255	255	0.541	220
1.09749	0.548745	0.301121	255	255	255	0.946	221
1.173189	0.586594	0.344093	255	255	255	1.081	222
1.710528	0.855264	0.731476	255	255	255	2.298	223
0.829953	0.414977	0.172206	255	255	255	0.541	224
0.829953	0.414977	0.172206	255	255	255	0.541	225
1.173189	0.586594	0.344093	255	255	255	1.081	226
0.829953	0.414977	0.172206	255	255	255	0.541	227
0.927745	0.463872	0.215177	255	255	255	0.676	228
0.927745	0.463872	0.215177	255	255	255	0.676	229
1.173189	0.586594	0.344093	255	255	255	1.081	230
1.244291	0.622145	0.387065	255	255	255	1.216	231
0.829953	0.414977	0.172206	255	255	255	0.541	232
1.016168	0.508084	0.258149	255	255	255	0.811	233
0.927745	0.463872	0.215177	255	255	255	0.676	234
1.437078	0.718539	0.516299	255	255	255	1.622	235
0.829953	0.414977	0.172206	255	255	255	0.541	236
1.016168	0.508084	0.258149	255	255	255	0.811	237
0.829953	0.414977	0.172206	255	255	255	0.541	238
0.829953	0.414977	0.172206	255	255	255	0.541	239
1.437078	0.718539	0.516299	255	255	255	1.622	240
0.829953	0.414977	0.172206	255	255	255	0.541	241
1.09749	0.548745	0.301121	255	255	255	0.946	242
0.829953	0.414977	0.172206	255	255	255	0.541	243
0.927745	0.463872	0.215177	255	255	255	0.676	244
0.829953	0.414977	0.172206	255	255	255	0.541	245
1.312029	0.656014	0.430355	255	255	255	1.352	246
0.829953	0.414977	0.172206	255	255	255	0.541	247
1.244291	0.622145	0.387065	255	255	255	1.216	248
0.829953	0.414977	0.172206	255	255	255	0.541	249
0.829953	0.414977	0.172206	255	255	255	0.541	250
0.927745	0.463872	0.215177	255	255	255	0.676	251
0.829953	0.414977	0.172206	255	255	255	0.541	252
0.829953	0.414977	0.172206	255	255	255	0.541	253
1.016168	0.508084	0.258149	255	255	255	0.811	254
0.829953	0.414977	0.172206	255	255	255	0.541	255
1.016168	0.508084	0.258149	255	255	255	0.811	256
1.244291	0.622145	0.387065	255	255	255	1.216	257
0.829953	0.414977	0.172206	255	255	255	0.541	258
0.829953	0.414977	0.172206	255	255	255	0.541	259
1.016168	0.508084	0.258149	255	255	255	0.811	260
1.710528	0.855264	0.731476	255	255	255	2.298	261
0.927745	0.463872	0.215177	255	255	255	0.676	262

1.552085	0.776043	0.602242	255	255	255	1.892	263
0.829953	0.414977	0.172206	255	255	255	0.541	264
1.09749	0.548745	0.301121	255	255	255	0.946	265
0.829953	0.414977	0.172206	255	255	255	0.541	266
1.495688	0.747844	0.55927	255	255	255	1.757	267
1.312029	0.656014	0.430355	255	255	255	1.352	268
0.829953	0.414977	0.172206	255	255	255	0.541	269
1.016168	0.508084	0.258149	255	255	255	0.811	270
0.829953	0.414977	0.172206	255	255	255	0.541	271
0.829953	0.414977	0.172206	255	255	255	0.541	272
0.829953	0.414977	0.172206	255	255	255	0.541	273
0.829953	0.414977	0.172206	255	255	255	0.541	274
0.829953	0.414977	0.172206	255	255	255	0.541	275
0.829953	0.414977	0.172206	255	255	255	0.541	276
0.927745	0.463872	0.215177	255	255	255	0.676	277
0.829953	0.414977	0.172206	255	255	255	0.541	278
1.016168	0.508084	0.258149	255	255	255	0.811	279
1.09749	0.548745	0.301121	255	255	255	0.946	280
1.312029	0.656014	0.430355	255	255	255	1.352	281
1.437078	0.718539	0.516299	255	255	255	1.622	282
0.829953	0.414977	0.172206	255	255	255	0.541	283
1.659523	0.829762	0.688504	255	255	255	2.163	284
1.244291	0.622145	0.387065	255	255	255	1.216	285
1.173189	0.586594	0.344093	255	255	255	1.081	286
1.173189	0.586594	0.344093	255	255	255	1.081	287
0.829953	0.414977	0.172206	255	255	255	0.541	288
0.829953	0.414977	0.172206	255	255	255	0.541	289
0.829953	0.414977	0.172206	255	255	255	0.541	290
0.927745	0.463872	0.215177	255	255	255	0.676	291
1.375975	0.687988	0.473327	255	255	255	1.487	292
0.829953	0.414977	0.172206	255	255	255	0.541	293
1.173189	0.586594	0.344093	255	255	255	1.081	294
1.173189	0.586594	0.344093	255	255	255	1.081	295
1.016168	0.508084	0.258149	255	255	255	0.811	296
0.829953	0.414977	0.172206	255	255	255	0.541	297
0.829953	0.414977	0.172206	255	255	255	0.541	298
1.437078	0.718539	0.516299	255	255	255	1.622	299
1.173189	0.586594	0.344093	255	255	255	1.081	300
0.829953	0.414977	0.172206	255	255	255	0.541	301
0.829953	0.414977	0.172206	255	255	255	0.541	302
1.173189	0.586594	0.344093	255	255	255	1.081	303
0.829953	0.414977	0.172206	255	255	255	0.541	304
1.016168	0.508084	0.258149	255	255	255	0.811	305
0.927745	0.463872	0.215177	255	255	255	0.676	306
1.173189	0.586594	0.344093	255	255	255	1.081	307
1.09749	0.548745	0.301121	255	255	255	0.946	308
1.375975	0.687988	0.473327	255	255	255	1.487	309
1.244291	0.622145	0.387065	255	255	255	1.216	310
1.312029	0.656014	0.430355	255	255	255	1.352	311
1.375975	0.687988	0.473327	255	255	255	1.487	312

0.829953	0.414977	0.172206	255	255	255	0.541		313
0.829953	0.414977	0.172206	255	255	255	0.541		314
0.829953	0.414977	0.172206	255	255	255	0.541		315
1.173189	0.586594	0.344093	255	255	255	1.081		316
1.09749	0.548745	0.301121	255	255	255	0.946		317
0.829953	0.414977	0.172206	255	255	255	0.541		318
0.927745	0.463872	0.215177	255	255	255	0.676		319
0.829953	0.414977	0.172206	255	255	255	0.541		320
1.09749	0.548745	0.301121	255	255	255	0.946		321
0.927745	0.463872	0.215177	255	255	255	0.676		322
0.927745	0.463872	0.215177	255	255	255	0.676		323
0.829953	0.414977	0.172206	255	255	255	0.541		324
2.032336	1.016168	1.032597	255	255	255	3.244		325
0.829953	0.414977	0.172206	255	255	255	0.541		326
1.244291	0.622145	0.387065	255	255	255	1.216		327
1.016168	0.508084	0.258149	255	255	255	0.811		328
1.173189	0.586594	0.344093	255	255	255	1.081		329
1.09749	0.548745	0.301121	255	255	255	0.946		330
0.829953	0.414977	0.172206	255	255	255	0.541		331
0.829953	0.414977	0.172206	255	255	255	0.541		332
1.016168	0.508084	0.258149	255	255	255	0.811		333
1.09749	0.548745	0.301121	255	255	255	0.946		334
0.927745	0.463872	0.215177	255	255	255	0.676		335
1.247867	0.623933	0.389293	255	255	255	1.223	Mean	336
1.651448	0.825724	0.68182	0	0	0	2.142	SD	337
0.829953	0.414977	0.172206	255	255	255	0.541	Min	338
3.411738	1.705869	2.909989	255	255	255	9.142	Max	339

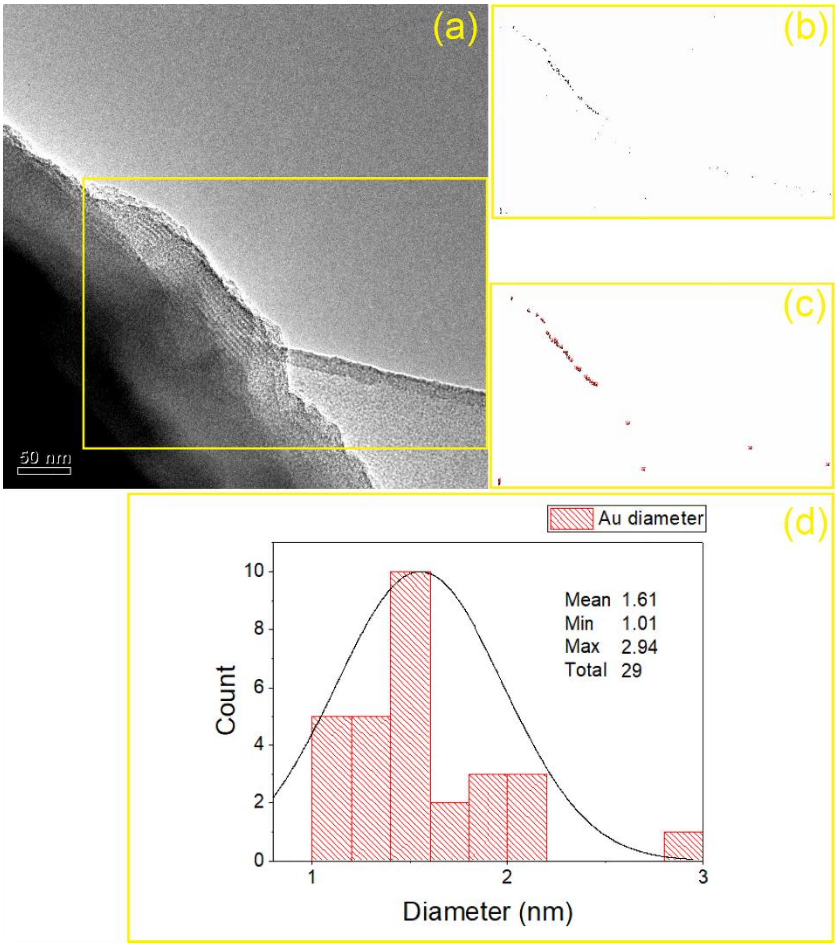

Figure S4. (a) Represent the HRTEM image of Figure 3(c) in the text of 1–Au–SBA–11, (b) is the adjusted contrast of (a), (c) the chosen particles numbered from (b), and (d) the produced particles size distribution.

Figure S4 (a) represents the selected area of image 3(b) in the text highlighted in yellow. While S4 (b) shows the Au clusters clearer after adjusting the contrast. S4 (c) showing the chosen Au particles and their labels of the selected area. S4 (d) is the produced histogram of the selected Au clusters. These selected Au clusters and their parameters are listed in Table S2.

Table S2 showing the numbered Au particles and their parameters.

d (nm)	r	r^2	Max	Min	Mean	Area	Label
1.511354	0.755677	0.571048	255	255	255	1.794	1
1.511354	0.755677	0.571048	255	255	255	1.794	2
1.592974	0.796487	0.634392	255	255	255	1.993	3
1.007359	0.503679	0.253693	255	255	255	0.797	4
1.884765	0.942382	0.888085	255	255	255	2.79	5
2.13708	1.06854	1.141778	255	255	255	3.587	6
1.884765	0.942382	0.888085	255	255	255	2.79	7
1.234016	0.617008	0.380699	255	255	255	1.196	8
1.424621	0.71231	0.507386	255	255	255	1.594	9
2.19585	1.097925	1.20544	255	255	255	3.787	10
1.424621	0.71231	0.507386	255	255	255	1.594	11
1.816305	0.908152	0.824741	255	255	255	2.591	12
2.937256	1.468628	2.156868	255	255	255	6.776	13
1.511354	0.755677	0.571048	255	255	255	1.794	14
1.234016	0.617008	0.380699	255	255	255	1.196	15
1.511354	0.755677	0.571048	255	255	255	1.794	16
1.745162	0.872581	0.761397	255	255	255	2.392	17
1.12612	0.56306	0.317037	255	255	255	0.996	18
1.511354	0.755677	0.571048	255	255	255	1.794	19
2.076953	1.038477	1.078434	255	255	255	3.388	20
1.234016	0.617008	0.380699	255	255	255	1.196	21
1.592974	0.796487	0.634392	255	255	255	1.993	22
1.33273	0.666365	0.444042	255	255	255	1.395	23
1.007359	0.503679	0.253693	255	255	255	0.797	24
1.007359	0.503679	0.253693	255	255	255	0.797	25
1.007359	0.503679	0.253693	255	255	255	0.797	26
1.234016	0.617008	0.380699	255	255	255	1.196	27
1.745162	0.872581	0.761397	255	255	255	2.392	28
1.511354	0.755677	0.571048	255	255	255	1.794	29
1.606504	0.803252	0.645214	255	255	255	2.027	30
1.253466	0.626733	0.392794	0	0	0	1.234	31
1.007359	0.503679	0.253693	255	255	255	0.797	32
2.937256	1.468628	2.156868	255	255	255	6.776	33

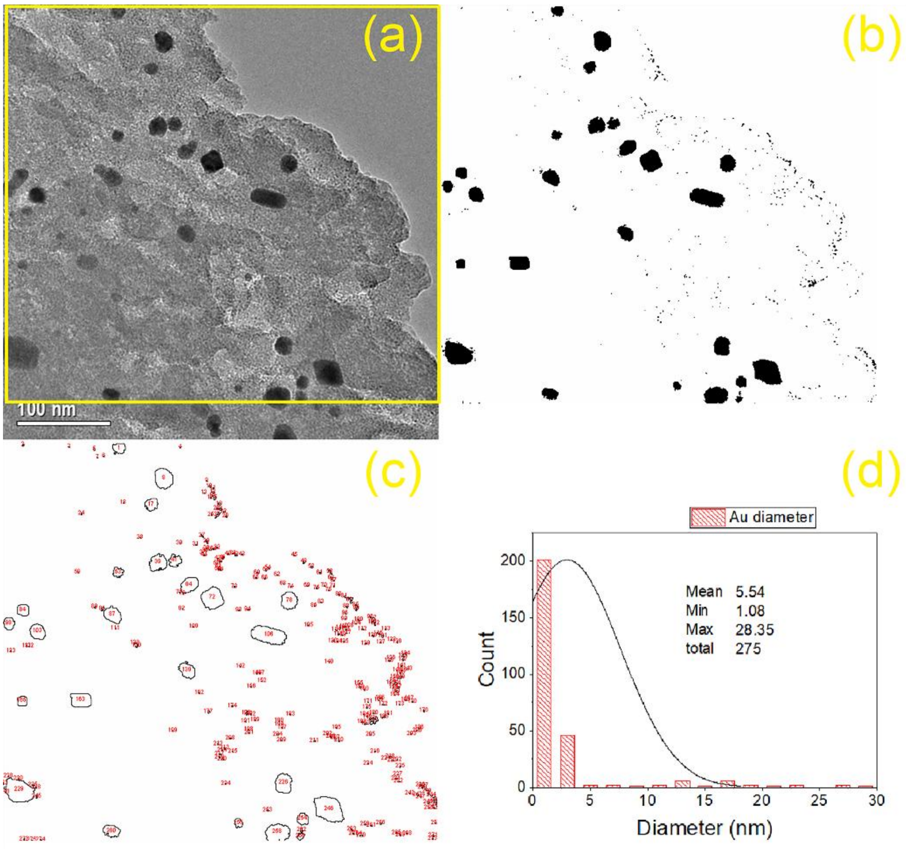

Figure S5. (a) Represent the HRTEM image of Figure 4(e) in the text, of 2–Au–SBA–11 (b) is the adjusted contrast of (a), (c) the chosen particles numbered from (b) and (d) the produced particles size distribution.

Figure S5 (a) represents the selected area of image 4(e) in the text highlighted in yellow. While S5 (b) shows the Au clusters clearer after adjusting the contrast. S5 (c) showing the chosen Au particles and their labels of the selected area. S5 (d) is the produced histogram of the selected Au clusters. These selected Au clusters and their parameters are listed in Table S3.

Table S3 shows the labeled Au particles and their parameters.

d (nm)	r	r^2	Max	Min	Mean	Area	Label
12.26601	6.133003	37.61372	255	255	255	118.167	1
1.700823	0.850412	0.7232	255	255	255	2.272	2
1.075814	0.537907	0.289344	255	255	255	0.909	3
1.075814	0.537907	0.289344	255	255	255	0.909	4
1.863364	0.931682	0.868031	255	255	255	2.727	5
1.075814	0.537907	0.289344	255	255	255	0.909	6
1.075814	0.537907	0.289344	255	255	255	0.909	7
20.49672	10.24836	105.0289	255	255	255	329.958	8
1.075814	0.537907	0.289344	255	255	255	0.909	9
2.012505	1.006252	1.012544	255	255	255	3.181	10
1.317355	0.658678	0.433856	255	255	255	1.363	11
2.282006	1.141003	1.301887	255	255	255	4.09	12
1.075814	0.537907	0.289344	255	255	255	0.909	13
1.075814	0.537907	0.289344	255	255	255	0.909	14
2.946129	1.473064	2.169918	255	255	255	6.817	15
1.317355	0.658678	0.433856	255	255	255	1.363	16
13.10976	6.554878	42.96642	255	255	255	134.983	17
1.075814	0.537907	0.289344	255	255	255	0.909	18
2.405592	1.202796	1.446718	255	255	255	4.545	19
1.317355	0.658678	0.433856	255	255	255	1.363	20
2.635194	1.317597	1.736062	255	255	255	5.454	21
1.52143	0.760715	0.578687	255	255	255	1.818	22
3.56807	1.784035	3.182781	255	255	255	9.999	23
1.700823	0.850412	0.7232	255	255	255	2.272	24
1.075814	0.537907	0.289344	255	255	255	0.909	25
1.863364	0.931682	0.868031	255	255	255	2.727	26
2.012505	1.006252	1.012544	255	255	255	3.181	27
1.075814	0.537907	0.289344	255	255	255	0.909	28
2.52288	1.26144	1.591231	255	255	255	4.999	29
1.075814	0.537907	0.289344	255	255	255	0.909	30
1.075814	0.537907	0.289344	255	255	255	0.909	31
1.317355	0.658678	0.433856	255	255	255	1.363	32
1.317355	0.658678	0.433856	255	255	255	1.363	33
1.317355	0.658678	0.433856	255	255	255	1.363	34
1.700823	0.850412	0.7232	255	255	255	2.272	35
1.075814	0.537907	0.289344	255	255	255	0.909	36
1.317355	0.658678	0.433856	255	255	255	1.363	37
1.700823	0.850412	0.7232	255	255	255	2.272	38
17.92103	8.960514	80.2908	255	255	255	252.241	39
1.52143	0.760715	0.578687	255	255	255	1.818	40
12.5689	6.284449	39.4943	255	255	255	124.075	41
2.282006	1.141003	1.301887	255	255	255	4.09	42
1.317355	0.658678	0.433856	255	255	255	1.363	43
1.317355	0.658678	0.433856	255	255	255	1.363	44
1.075814	0.537907	0.289344	255	255	255	0.909	45
2.405592	1.202796	1.446718	255	255	255	4.545	46
1.075814	0.537907	0.289344	255	255	255	0.909	47

2.742681	1.371341	1.880575	255	255	255	5.908	48
2.012505	1.006252	1.012544	255	255	255	3.181	49
1.075814	0.537907	0.289344	255	255	255	0.909	50
1.075814	0.537907	0.289344	255	255	255	0.909	51
2.282006	1.141003	1.301887	255	255	255	4.09	52
10.43028	5.215139	27.19767	255	255	255	85.444	53
1.863364	0.931682	0.868031	255	255	255	2.727	54
1.075814	0.537907	0.289344	255	255	255	0.909	55
1.075814	0.537907	0.289344	255	255	255	0.909	56
1.700823	0.850412	0.7232	255	255	255	2.272	57
3.04286	1.52143	2.314749	255	255	255	7.272	58
1.075814	0.537907	0.289344	255	255	255	0.909	59
1.700823	0.850412	0.7232	255	255	255	2.272	60
1.52143	0.760715	0.578687	255	255	255	1.818	61
1.075814	0.537907	0.289344	255	255	255	0.909	62
2.52288	1.26144	1.591231	255	255	255	4.999	63
17.2799	8.639951	74.64876	255	255	255	234.516	64
1.52143	0.760715	0.578687	255	255	255	1.818	65
1.317355	0.658678	0.433856	255	255	255	1.363	66
1.317355	0.658678	0.433856	255	255	255	1.363	67
1.075814	0.537907	0.289344	255	255	255	0.909	68
1.075814	0.537907	0.289344	255	255	255	0.909	69
1.075814	0.537907	0.289344	255	255	255	0.909	70
1.075814	0.537907	0.289344	255	255	255	0.909	71
22.84649	11.42324	130.4905	255	255	255	409.948	72
1.52143	0.760715	0.578687	255	255	255	1.818	73
1.317355	0.658678	0.433856	255	255	255	1.363	74
1.52143	0.760715	0.578687	255	255	255	1.818	75
1.075814	0.537907	0.289344	255	255	255	0.909	76
1.317355	0.658678	0.433856	255	255	255	1.363	77
17.8401	8.92005	79.56729	255	255	255	249.968	78
1.075814	0.537907	0.289344	255	255	255	0.909	79
1.700823	0.850412	0.7232	255	255	255	2.272	80
2.012505	1.006252	1.012544	255	255	255	3.181	81
4.56429	2.282145	5.208186	255	255	255	16.362	82
1.075814	0.537907	0.289344	255	255	255	0.909	83
12.84217	6.421087	41.23036	255	255	255	129.529	84
1.317355	0.658678	0.433856	255	255	255	1.363	85
1.075814	0.537907	0.289344	255	255	255	0.909	86
17.31336	8.65668	74.9381	255	255	255	235.425	87
1.863364	0.931682	0.868031	255	255	255	2.727	88
1.52143	0.760715	0.578687	255	255	255	1.818	89
2.846335	1.423168	2.025406	255	255	255	6.363	90
3.04286	1.52143	2.314749	255	255	255	7.272	91
1.075814	0.537907	0.289344	255	255	255	0.909	92
1.317355	0.658678	0.433856	255	255	255	1.363	93
1.317355	0.658678	0.433856	255	255	255	1.363	94
1.075814	0.537907	0.289344	255	255	255	0.909	95
1.52143	0.760715	0.578687	255	255	255	1.818	96
3.136407	1.568203	2.459262	255	255	255	7.726	97

12.26601	6.133003	37.61372	255	255	255	118.167	98
1.075814	0.537907	0.289344	255	255	255	0.909	99
1.863364	0.931682	0.868031	255	255	255	2.727	100
1.700823	0.850412	0.7232	255	255	255	2.272	101
2.151627	1.075814	1.157375	255	255	255	3.636	102
16.24423	8.122116	65.96877	255	255	255	207.247	103
1.075814	0.537907	0.289344	255	255	255	0.909	104
1.317355	0.658678	0.433856	255	255	255	1.363	105
26.14214	13.07107	170.8528	255	255	255	536.75	106
1.52143	0.760715	0.578687	255	255	255	1.818	107
1.075814	0.537907	0.289344	255	255	255	0.909	108
1.075814	0.537907	0.289344	255	255	255	0.909	109
1.317355	0.658678	0.433856	255	255	255	1.363	110
1.075814	0.537907	0.289344	255	255	255	0.909	111
1.700823	0.850412	0.7232	255	255	255	2.272	112
1.52143	0.760715	0.578687	255	255	255	1.818	113
1.700823	0.850412	0.7232	255	255	255	2.272	114
1.700823	0.850412	0.7232	255	255	255	2.272	115
2.282006	1.141003	1.301887	255	255	255	4.09	116
1.075814	0.537907	0.289344	255	255	255	0.909	117
1.700823	0.850412	0.7232	255	255	255	2.272	118
1.317355	0.658678	0.433856	255	255	255	1.363	119
1.700823	0.850412	0.7232	255	255	255	2.272	120
2.52288	1.26144	1.591231	255	255	255	4.999	121
1.317355	0.658678	0.433856	255	255	255	1.363	122
1.700823	0.850412	0.7232	255	255	255	2.272	123
1.317355	0.658678	0.433856	255	255	255	1.363	124
1.317355	0.658678	0.433856	255	255	255	1.363	125
1.317355	0.658678	0.433856	255	255	255	1.363	126
1.317355	0.658678	0.433856	255	255	255	1.363	127
1.700823	0.850412	0.7232	255	255	255	2.272	128
1.52143	0.760715	0.578687	255	255	255	1.818	129
2.282006	1.141003	1.301887	255	255	255	4.09	130
1.52143	0.760715	0.578687	255	255	255	1.818	131
1.075814	0.537907	0.289344	255	255	255	0.909	132
1.52143	0.760715	0.578687	255	255	255	1.818	133
1.863364	0.931682	0.868031	255	255	255	2.727	134
2.405592	1.202796	1.446718	255	255	255	4.545	135
1.075814	0.537907	0.289344	255	255	255	0.909	136
2.151627	1.075814	1.157375	255	255	255	3.636	137
1.863364	0.931682	0.868031	255	255	255	2.727	138
16.22639	8.113195	65.82394	255	255	255	206.792	139
1.075814	0.537907	0.289344	255	255	255	0.909	140
1.52143	0.760715	0.578687	255	255	255	1.818	141
1.075814	0.537907	0.289344	255	255	255	0.909	142
1.52143	0.760715	0.578687	255	255	255	1.818	143
1.52143	0.760715	0.578687	255	255	255	1.818	144
1.317355	0.658678	0.433856	255	255	255	1.363	145
1.317355	0.658678	0.433856	255	255	255	1.363	146
1.317355	0.658678	0.433856	255	255	255	1.363	147

1.075814	0.537907	0.289344	255	255	255	0.909	148
2.946129	1.473064	2.169918	255	255	255	6.817	149
1.075814	0.537907	0.289344	255	255	255	0.909	150
1.700823	0.850412	0.7232	255	255	255	2.272	151
1.075814	0.537907	0.289344	255	255	255	0.909	152
1.52143	0.760715	0.578687	255	255	255	1.818	153
2.742681	1.371341	1.880575	255	255	255	5.908	154
1.52143	0.760715	0.578687	255	255	255	1.818	155
1.317355	0.658678	0.433856	255	255	255	1.363	156
2.635194	1.317597	1.736062	255	255	255	5.454	157
1.863364	0.931682	0.868031	255	255	255	2.727	158
1.075814	0.537907	0.289344	255	255	255	0.909	159
1.317355	0.658678	0.433856	255	255	255	1.363	160
2.405592	1.202796	1.446718	255	255	255	4.545	161
1.317355	0.658678	0.433856	255	255	255	1.363	162
19.10869	9.554347	91.28555	255	255	255	286.782	163
1.075814	0.537907	0.289344	255	255	255	0.909	164
10.73106	5.365529	28.7889	255	255	255	90.443	165
1.52143	0.760715	0.578687	255	255	255	1.818	166
1.075814	0.537907	0.289344	255	255	255	0.909	167
1.52143	0.760715	0.578687	255	255	255	1.818	168
1.075814	0.537907	0.289344	255	255	255	0.909	169
1.075814	0.537907	0.289344	255	255	255	0.909	170
1.075814	0.537907	0.289344	255	255	255	0.909	171
1.52143	0.760715	0.578687	255	255	255	1.818	172
1.075814	0.537907	0.289344	255	255	255	0.909	173
1.075814	0.537907	0.289344	255	255	255	0.909	174
1.075814	0.537907	0.289344	255	255	255	0.909	175
1.317355	0.658678	0.433856	255	255	255	1.363	176
2.282006	1.141003	1.301887	255	255	255	4.09	177
2.151627	1.075814	1.157375	255	255	255	3.636	178
1.52143	0.760715	0.578687	255	255	255	1.818	179
1.075814	0.537907	0.289344	255	255	255	0.909	180
1.317355	0.658678	0.433856	255	255	255	1.363	181
1.52143	0.760715	0.578687	255	255	255	1.818	182
1.317355	0.658678	0.433856	255	255	255	1.363	183
1.075814	0.537907	0.289344	255	255	255	0.909	184
1.317355	0.658678	0.433856	255	255	255	1.363	185
2.52288	1.26144	1.591231	255	255	255	4.999	186
6.318923	3.159462	9.982198	255	255	255	31.36	187
2.52288	1.26144	1.591231	255	255	255	4.999	188
1.075814	0.537907	0.289344	255	255	255	0.909	189
1.075814	0.537907	0.289344	255	255	255	0.909	190
1.075814	0.537907	0.289344	255	255	255	0.909	191
1.700823	0.850412	0.7232	255	255	255	2.272	192
2.012505	1.006252	1.012544	255	255	255	3.181	193
1.075814	0.537907	0.289344	255	255	255	0.909	194
1.317355	0.658678	0.433856	255	255	255	1.363	195
1.863364	0.931682	0.868031	255	255	255	2.727	196
1.075814	0.537907	0.289344	255	255	255	0.909	197

1.317355	0.658678	0.433856	255	255	255	1.363	198
1.075814	0.537907	0.289344	255	255	255	0.909	199
1.52143	0.760715	0.578687	255	255	255	1.818	200
1.075814	0.537907	0.289344	255	255	255	0.909	201
1.317355	0.658678	0.433856	255	255	255	1.363	202
1.075814	0.537907	0.289344	255	255	255	0.909	203
1.700823	0.850412	0.7232	255	255	255	2.272	204
1.075814	0.537907	0.289344	255	255	255	0.909	205
1.700823	0.850412	0.7232	255	255	255	2.272	206
2.151627	1.075814	1.157375	255	255	255	3.636	207
1.52143	0.760715	0.578687	255	255	255	1.818	208
1.075814	0.537907	0.289344	255	255	255	0.909	209
1.700823	0.850412	0.7232	255	255	255	2.272	210
1.52143	0.760715	0.578687	255	255	255	1.818	211
1.317355	0.658678	0.433856	255	255	255	1.363	212
1.700823	0.850412	0.7232	255	255	255	2.272	213
1.317355	0.658678	0.433856	255	255	255	1.363	214
1.075814	0.537907	0.289344	255	255	255	0.909	215
1.317355	0.658678	0.433856	255	255	255	1.363	216
1.863364	0.931682	0.868031	255	255	255	2.727	217
2.282006	1.141003	1.301887	255	255	255	4.09	218
1.52143	0.760715	0.578687	255	255	255	1.818	219
1.863364	0.931682	0.868031	255	255	255	2.727	220
1.075814	0.537907	0.289344	255	255	255	0.909	221
1.075814	0.537907	0.289344	255	255	255	0.909	222
1.700823	0.850412	0.7232	255	255	255	2.272	223
1.317355	0.658678	0.433856	255	255	255	1.363	224
1.52143	0.760715	0.578687	255	255	255	1.818	225
19.28954	9.644771	93.02161	255	255	255	292.236	226
1.075814	0.537907	0.289344	255	255	255	0.909	227
1.317355	0.658678	0.433856	255	255	255	1.363	228
27.29012	13.64506	186.1877	255	255	255	584.926	229
1.075814	0.537907	0.289344	255	255	255	0.909	230
1.863364	0.931682	0.868031	255	255	255	2.727	231
1.317355	0.658678	0.433856	255	255	255	1.363	232
1.317355	0.658678	0.433856	255	255	255	1.363	233
1.317355	0.658678	0.433856	255	255	255	1.363	234
2.151627	1.075814	1.157375	255	255	255	3.636	235
2.151627	1.075814	1.157375	255	255	255	3.636	236
1.863364	0.931682	0.868031	255	255	255	2.727	237
1.317355	0.658678	0.433856	255	255	255	1.363	238
1.317355	0.658678	0.433856	255	255	255	1.363	239
3.803492	1.901746	3.616637	255	255	255	11.362	240
1.075814	0.537907	0.289344	255	255	255	0.909	241
1.317355	0.658678	0.433856	255	255	255	1.363	242
2.405592	1.202796	1.446718	255	255	255	4.545	243
1.863364	0.931682	0.868031	255	255	255	2.727	244
1.863364	0.931682	0.868031	255	255	255	2.727	245
28.35094	14.17547	200.9439	255	255	255	631.284	246
1.075814	0.537907	0.289344	255	255	255	0.909	247

4.689362	2.344681	5.49753	255	255	255	17.271	248
2.151627	1.075814	1.157375	255	255	255	3.636	249
2.635194	1.317597	1.736062	255	255	255	5.454	250
1.075814	0.537907	0.289344	255	255	255	0.909	251
1.52143	0.760715	0.578687	255	255	255	1.818	252
1.52143	0.760715	0.578687	255	255	255	1.818	253
12.14746	6.073731	36.89021	255	255	255	115.894	254
8.936236	4.468118	19.96408	255	255	255	62.719	255
2.012505	1.006252	1.012544	255	255	255	3.181	256
1.52143	0.760715	0.578687	255	255	255	1.818	257
22.11283	11.05642	122.2444	255	255	255	384.042	258
1.52143	0.760715	0.578687	255	255	255	1.818	259
14.75064	7.37532	54.39534	255	255	255	170.888	260
1.700823	0.850412	0.7232	255	255	255	2.272	261
3.227441	1.61372	2.604093	255	255	255	8.181	262
1.700823	0.850412	0.7232	255	255	255	2.272	263
2.012505	1.006252	1.012544	255	255	255	3.181	264
1.317355	0.658678	0.433856	255	255	255	1.363	265
6.930398	3.465199	12.0076	255	255	255	37.723	266
1.317355	0.658678	0.433856	255	255	255	1.363	267
1.317355	0.658678	0.433856	255	255	255	1.363	268
1.52143	0.760715	0.578687	255	255	255	1.818	269
2.151627	1.075814	1.157375	255	255	255	3.636	270
1.317355	0.658678	0.433856	255	255	255	1.363	271
1.52143	0.760715	0.578687	255	255	255	1.818	272
1.52143	0.760715	0.578687	255	255	255	1.818	273
1.52143	0.760715	0.578687	255	255	255	1.818	274
1.075814	0.537907	0.289344	255	255	255	0.909	275
5.537112	2.768556	7.664902	255	255	255	24.08	276
10.33561	5.167804	26.7062	0	0	0	83.9	277
1.075814	0.537907	0.289344	255	255	255	0.909	278
28.35094	14.17547	200.9439	255	255	255	631.284	279

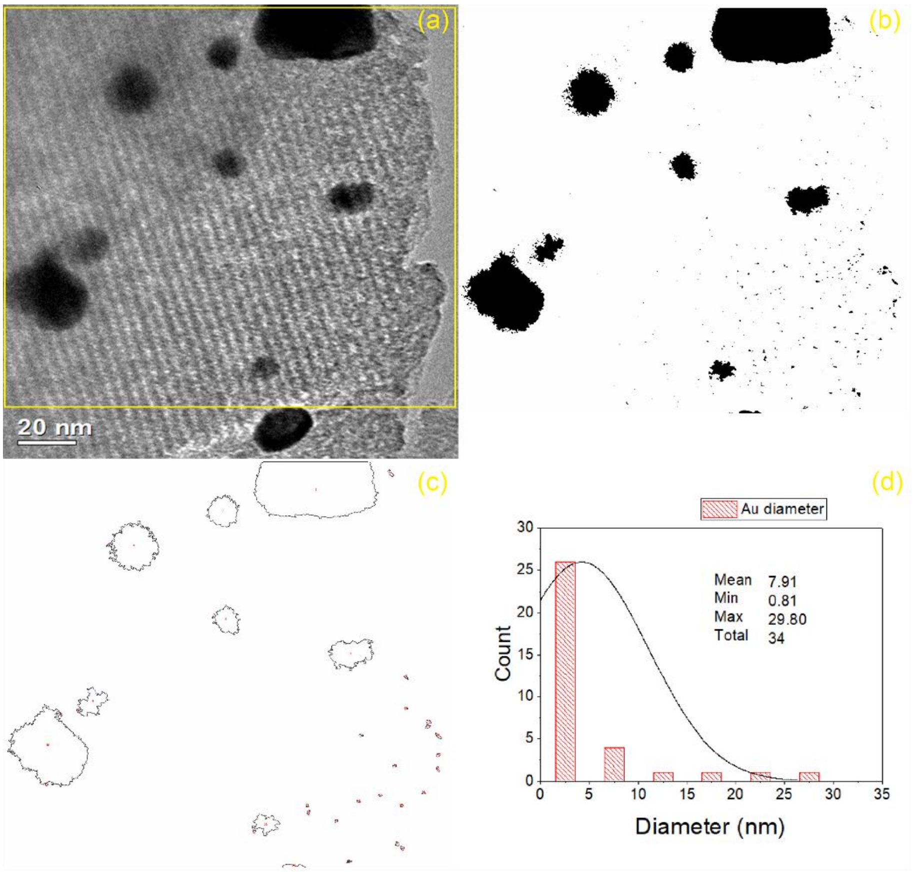

Figure S6. (a) Represent the HRTEM image of Figure 4(f) of 2–Au–SBA–11 in the text, (b) is the adjusted contrast of (a), (c) the chosen particles numbered from (b) and (d) the produced particles size distribution.

Figure S6 (a) represents the selected area of image 4(f) in the text highlighted in yellow. While S6 (b) shows the Au clusters clearer after adjusting the contrast. S6 (c) showing the chosen Au particles and their labels of the selected area. S6 (d) is the produced histogram of the selected Au clusters. These selected Au clusters and their parameters are listed in Table S4.

Table S4 shows the numbered Au particles and their parameters.

d (nm)	r	r^2	Max	Min	Mean	Area	Label
29.80299	14.9015	222.0546	255	255	255	697.605	1
1.472956	0.736478	0.5424	255	255	255	1.704	2
9.952151	4.976075	24.76133	255	255	255	77.79	3
15.25413	7.627063	58.17209	255	255	255	182.753	4
1.253974	0.626987	0.393113	255	255	255	1.235	5
8.287098	4.143549	17.169	255	255	255	53.938	6
11.40986	5.70493	32.54623	255	255	255	102.247	7
1.149065	0.574532	0.330087	255	255	255	1.037	8
8.09324	4.04662	16.37513	255	255	255	51.444	9
23.7175	11.85875	140.6299	255	255	255	441.802	10
0.841381	0.42069	0.17698	255	255	255	0.556	11
0.822247	0.411124	0.169023	255	255	255	0.531	12
0.841381	0.42069	0.17698	255	255	255	0.556	13
1.412954	0.706477	0.49911	255	255	255	1.568	14
1.33273	0.666365	0.444042	255	255	255	1.395	15
1.429528	0.714764	0.510887	255	255	255	1.605	16
0.954798	0.477399	0.22791	255	255	255	0.716	17
1.215302	0.607651	0.369239	255	255	255	1.16	18
1.018671	0.509335	0.259423	255	255	255	0.815	19
1.063913	0.531956	0.282977	255	255	255	0.889	20
0.831486	0.415743	0.172842	255	255	255	0.543	21
0.850412	0.425206	0.1808	255	255	255	0.568	22
0.812903	0.406452	0.165203	255	255	255	0.519	23
0.877673	0.438837	0.192577	255	255	255	0.605	24
0.859348	0.429674	0.18462	255	255	255	0.58	25
0.850412	0.425206	0.1808	255	255	255	0.568	26
1.121588	0.560794	0.31449	255	255	255	0.988	27
6.674437	3.337218	11.13703	255	255	255	34.988	28
1.010514	0.505257	0.255285	255	255	255	0.802	29
1.010514	0.505257	0.255285	255	255	255	0.802	30
1.175899	0.587949	0.345685	255	255	255	1.086	31
1.314937	0.657468	0.432265	255	255	255	1.358	32
1.002925	0.501463	0.251465	255	255	255	0.79	33
2.795102	1.397551	1.953149	255	255	255	6.136	34
7.912103	3.956051	15.65034	255	255	255	49.167	35
13.40042	6.700212	44.89283	0	0	0	141.035	36
0.812903	0.406452	0.165203	255	255	255	0.519	37
29.80299	14.9015	222.0546	255	255	255	697.605	38

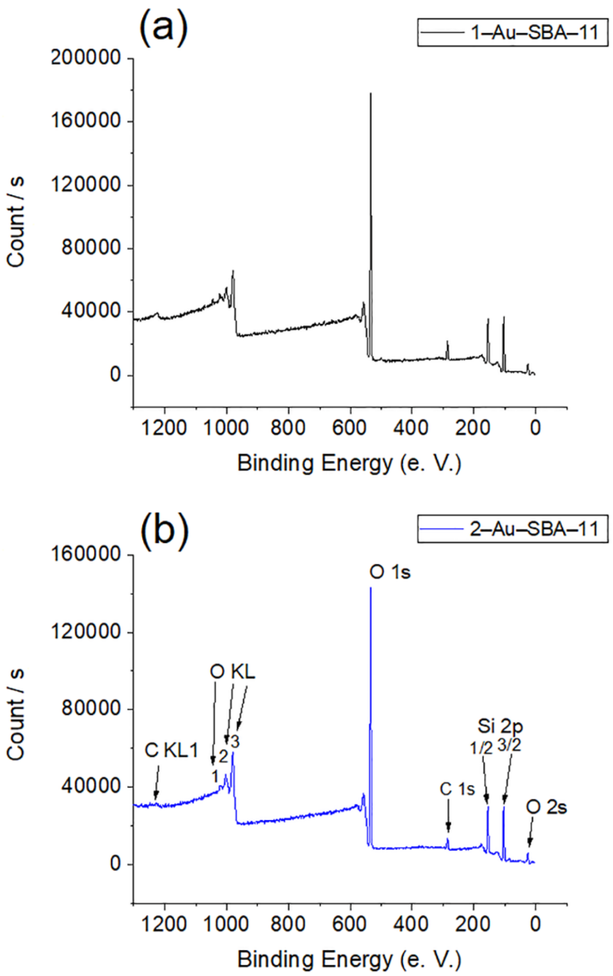

Figure S7. full range XPS survey of (a) 1–Au–SBA–11, (b) 2–Au–SBA–11.

Figure S 7 shows the full XPS survey of both materials, (a) 1–Au–SBA–11, (b) 2–Au–SBA–11. All elements are identified in Figure S7(b), XPS are similar for both materials. There is no foreigner element detected and the carbon atomic % were less than 2 at. % for both materials mainly adventitious carbon.

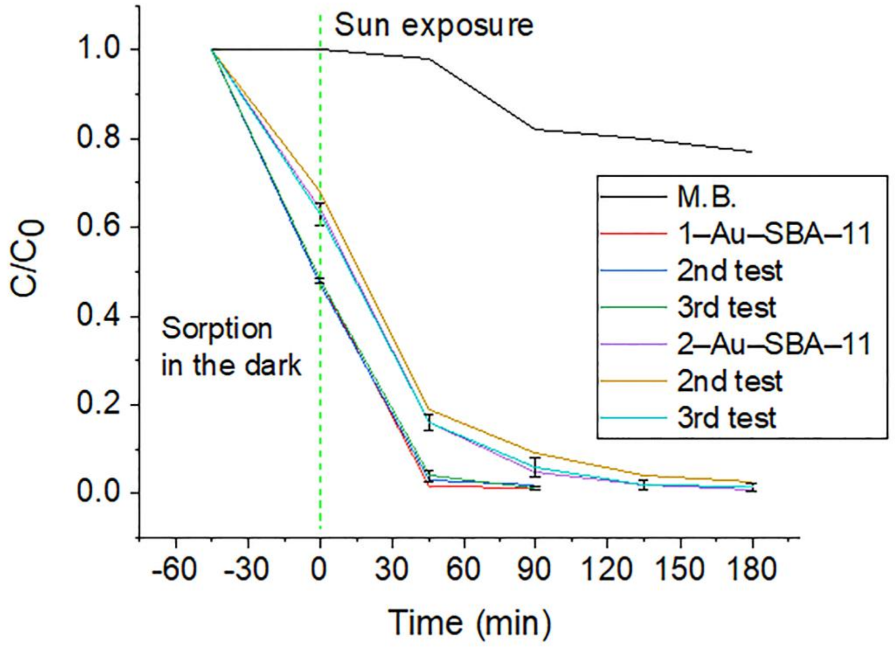

Figure S8. Photocatalytic behavior of M.B. solution with and without the catalysts.

Figure S8 is similar to Figure (9a) in the main text showing the photocatalytic behavior of M.B. solution with and without the catalysts. Additionally, Figure S8 includes the standard deviation bars.
